# Genome‐wide association study and KASP marker development for starch quality traits in wheat

**DOI:** 10.1002/tpg2.20514

**Published:** 2024-09-29

**Authors:** Yousheng Tian, Pengpeng Liu, Xin Zhang, Yichen Liu, Dezhen Kong, Yingbin Nie, Hongjun Xu, Xinnian Han, Wei Sang, Weihua Li

**Affiliations:** ^1^ Department of Administrative Management Xinjiang Academy of Agriculture and Reclamation Sciences Shihezi China; ^2^ Institute of Crop Science Xinjiang Academy of Agriculture and Reclamation Sciences Shihezi China; ^3^ Key Laboratory of Xinjiang Production and Construction Corps for Cereal Quality Research and Genetic Improvement Xinjiang Academy of Agriculture and Reclamation Sciences Shihezi China; ^4^ The Key Laboratory of the Oasis Ecological Agriculture, College of Agriculture Shihezi University Shihezi China

## Abstract

Starch is the main component of wheat (*Triticum aestivum* L.) flour, and its quality directly affects the processing quality of the final product. To investigate the genetic basis of starch, this study assessed the starch quality traits of 341 winter wheat varieties/lines grown in Emin and Qitai during the years 2019–2020 and 2020–2021. A genome‐wide association study was conducted with the genotype data obtained from wheat 40K breeding chips using the mixed linear model. Wheat starch quality traits exhibited coefficients of variation ranging from 1.43% to 23.66% and broad‐sense heritabilities between 0.37 and 0.87. All traits followed an approximately normal distribution, except for T. There were highly significant correlations among starch quality traits, with the strongest correlation observed between final viscosity (FV) and trough viscosity (TV) (*r* = 0.748), followed by peak viscosity and breakdown (BD) (*r* = 0.679). Thirty‐four single‐nucleotide polymorphism markers significantly and stably associated with starch quality traits were identified, clustering in 31 genetic loci. These included one locus for TV, six loci for BD, three loci for FV, two loci for peak time (PT), 12 loci for T, five loci for falling number, and two loci for damaged starch. One PT‐related block of 410 kb was identified in the region of 596 Mb on chromosome 5A, where significant phenotypic differences were observed between different haplotypes. One Kompetitive allele‐specific PCR (KASP) marker for T was developed on chromosome 7B, and two KASP markers for BD were developed on chromosome 7A. Four candidate genes possibly affecting BD during grain development were identified on chromosome 7A, including *TraesCS7A02G225100.1*, *TraesCS7A02G225900.1*, *TraesCS7A02G226400.1*, and *TraesCS7A02G257100.1*. The results have significant implications for utilizing marker‐assisted selection in breeding to improve wheat starch quality.

## Core Ideas

- Thirty‐four single‐nucleotide polymorphism markers significantly and stably associated with starch quality traits were identified.
- One KASP marker for pasting temperature and two KASP markers for breakdown were developed to validate the accuracy of genome‐wide association study results.
- Quantitative real‐time polymerase chain reaction was used to analyze the expression of candidate genes in seeds of two extreme starch quality materials.

**Abbreviations**
BD: breakdown
BE: branching enzyme
BHLH: basic helix‐loop‐helix
BLUP: best linear unbiased prediction
DBE: debranching enzyme
DS: damaged starch
FN: falling number
FV: final viscosity
GBSS: granule‐bound starch synthase
GO: gene ontology
GWAS: genome‐wide association study
*h* ^2^
: broad‐sense heritability
KASP: Kompetitive allele‐specific PCR
KEGG: Kyoto encyclopedia of genes and genomes
LD: linkage disequilibrium
LMA: late maturity A‐amylase
MAS: marker‐assisted selection
MLM: mixed linear model
MTA: marker‐trait association
PT: peak time
PV: peak viscosity
QTL: quantitative trait locus
RVA: rapid viscosity analyzer
SB: setback
TV: trough viscosity

## 1 INTRODUCTION

Starch is the main storage component of wheat (*Triticum aestivum* L.) endosperm and also the primary component of wheat flour, accounting for 65%–75% of the dry weight of mature wheat seeds (G. X. Chen et al., 2016). Starch significantly impacts meal preparation, final product quality, nutritional value, and economic benefits. The starch quality traits of wheat flour, such as gelatinization characteristics, falling number (FN), and damaged starch (DS), are key indicators for evaluating food processing quality (Amiri et al., 2018; Crosbie, 1991; Kaur et al., 2016; J. J. Liu et al., 2003; Moiraghi et al., 2019; Muqaddasi et al., 2023). Assessing these starch quality traits during the breeding process was effective in estimating the final taste and cooking quality of wheat (Z. H. He et al., 2003; Z. H. He et al., 2004; León et al., 2006; Y. Zhang et al., 2004, 2005). However, conducting quality trait tests was resource‐intensive and nearly impossible in the early generations of the breeding process (Battenfield et al., 2018). Marker‐assisted selection (MAS) might offer advantages in selecting these quality traits. In the MAS method, it was essential to first identify the genetic loci and develop corresponding linkage markers (Adamski et al., 2020).

Gelatinization is the most important and significant change in wheat starch that occurs after heating. The rapid viscosity analyzer (RVA) indicates the gelatinization characteristics of starch by measuring the resistance of a flour‐water mixed batter to paddle agitation. Quantitative trait loci (QTLs) for RVA parameters have been identified on all chromosomes except 4D by previous researchers (Deng et al., 2014; Sun et al., 2008; Tian et al., 2022; Udall et al., 1999; Y. Zhang et al., 2009). The *Wx‐B1* gene encodes granule‐bound starch synthase (GBSS), leading to starch with higher peak viscosity (PV) and breakdown (BD) (Ram & Sharma, 2013). Previous studies have identified QTLs for starch gelatinization traits near *Wx‐B1* (Batey et al., 2002; McCartney et al., 2006). The *TRAESCS7D02G365900* gene, located on chromosome 7D, encodes a phosphorylase. This enzyme has been reported in the literature to be involved in starch synthesis and degradation (Mishra et al., 2016; Tickle et al., 2009). Tian et al. (2022) identified a QTL for PV nearby this gene.

FN measures the activity of the starch‐degrading enzyme α‐amylase in wheat flour (Perten, 1964). Varieties that germinate before harvest or have late maturity A‐amylase (LMA) will produce excessive α‐amylase (Lunn et al., 2001), resulting in a decrease in the FN. LMA is a genetic defect widely present in bread wheat and durum wheat (D. Mares & Mrva, 2008). The characteristic of LMA is that from the middle stage of grain development (25–30 days) to harvest maturity, the *α‐Amy‐1* gene encodes and synthesizes high isoelectric point α‐amylase (D. J. Mares & Gale, 1990). During the grinding process, some starch particles may suffer mechanical damage, which can disrupt the particle structure and produce DS. The degree of damage depends on the hardness of the wheat and the conditions and techniques used during the milling process (Delcour & Hoseney, 1994). Hard grains require more grinding energy than soft grains to convert endosperm into flour, resulting in more starch particles being physically damaged during the grinding process. The main difference in grain texture between soft wheat and hard wheat is determined by allelic differences in the Puroindoline genes *Pina* and *Pinb* located on the 5DS chromosome (Bhave & Morris, 2008a, 2008b; Morris, 2002).

Starch quality traits are quantitative traits controlled by multiple genes and are influenced by the environment (Y. Zhang et al., 2009). The traditional method for assessing starch quality requires expensive instruments and a large amount of labor. In the breeding process, using molecular markers directly for MAS is more effective than phenotype selection. Therefore, identifying the genetic basis of starch quality traits is crucial for enhancing wheat starch quality through MAS breeding.

## 2 MATERIALS AND METHODS

### 2.1 Plant materials and field trials

The association panel comprised 341 winter wheat varieties and outstanding self‐cultivated lines from various wheat regions in China (Table S1). The validation of KASP markers was conducted in a separate natural population of 200 winter wheat genotypes, referred to as the validation population in this article (Table S2). This population included domestic wheat varieties and self‐bred high‐generation lines that were distinct from the association panel.

The association panel was grown in Emin and Qitai County, Xinjiang, during the years 2019–2020 and 2020–2021 (referred to as 2020EM, 2020QT, 2021EM, and 2021QT, respectively). The validation panel was grown in Shihezi City, Xinjiang, during the years 2020–2021. The field experiment adopted an alpha Latin design with two replicates. Plant eight rows of each material, with a row length of 1.8 m and a row spacing of 25 cm. Field management was consistent with local practices. The MLU202 grinder was used for grinding, and flour samples were stored in sealed bags for testing starch quality traits.

### 2.2 Phenotype test

The gelatinization characteristics of flour were determined using RVA Techmaster (Newport Scientific). Three grams of flour were suspended in 25 mL of ddH_2_O and then placed in the RVA instrument. The temperature program was as follows: maintained at 50°C for 60 s, heated from 50°C to 95°C at a rate of 1°C/5 s, maintained at 95°C for 150 s, then cooled to 50°C at a rate of 1°C/5 s, and maintained at 50°C for 120 s. The variables recorded by the RVA instrument included PV, trough viscosity (TV), BD, final viscosity (FV), setback (SB), peak time (PT), and pasting temperature (T).

FN was measured using the FN1500 Fungal Falling Number Instrument (Perten) following the GB/T10361‐2008 method.

**Core Ideas**

- Thirty‐four single‐nucleotide polymorphism markers significantly and stably associated with starch quality traits were identified.
- One KASP marker for pasting temperature and two KASP markers for breakdown were developed to validate the accuracy of genome‐wide association study results.
- Quantitative real‐time polymerase chain reaction was used to analyze the expression of candidate genes in seeds of two extreme starch quality materials.

DS was measured using a damaged starch analyzer (Sdematic) following the AACC76‐31 method.

### 2.3 Genome‐wide association study

Genotyping was performed using the wheat GBTS 40K breeding chip (MolBreeding Biotech Ltd., https://www.molbreeding.com/index.php?m = home&c = Lists&a = index&tid = 46). Single‐nucleotide polymorphism (SNP) markers with minor allele frequencies below 5% and missing data exceeding 10% were excluded from the genome‐wide association study (GWAS). The structure analysis was conducted using the STRUCTURE v2.3.4 software (Pritchard et al., 2000). PCR and linkage disequilibrium (LD) analysis were performed using TASSEL 5.0 (Bradbury et al., 2007). The association panel was divided into two subgroups, with an LD decay distance of 4 Mb. These results were presented in another article (submitted but unpublished).

Utilized the mixed linear model (MLM) in TASSEL 5.0 (Bradbury et al., 2007) for GWAS. Used the group structure (Q matrix) and kinship matrix (K matrix) as covariates to prevent false positives. The genetic relationship coefficients between each pair of materials were estimated using the Loiselle algorithm in TASSEL 5.0 (Loiselle et al., 1995). A marker was considered significantly associated with the trait when the significance test reached *p* < 0.0001 (−log10(*p*) ≥ 4). Multiple SNPs significantly associated with a trait within an LD interval of a chromosome segment were referred to as a locus (Z. Liu et al., 2020). Loci identified across multiple environments were termed stable loci, while loci associated with various phenotypic traits were known as pleiotropic loci (Ghimire et al., 2022; Tian et al., 2022).

### 2.4 Haplotype analysis

Performed haplotype analysis on significantly associated loci using Haploview 4.2 software (Barrett et al., 2005). Blocks were generated by the Haploview software based on the confidence interval described by Gabriel et al. (2002).

### 2.5 Development and validation of KASP marker

SNPs that were significantly and consistently identified in multiple environments to develop KASP markers were selected. The online software Polymarker (http://www.polymarker.info/) was utilized to design two allele‐specific forward primers and one reverse common primer. Standard FAM tag (5′ GAAGGTGAGTCATGCT 3′) and HEX tag (5′ GAAGGTCGAGTCAACGGATT 3′) were attached to the 5′ ends of the two allele‐specific primers.

According to the descending heat cycle protocol described by the manufacturer (LGC Genomics), SNP genotyping and data analysis were performed using a 96‐well plate with an ABI7500 instrument (Santos et al., 2019). Applied a *t*‐test to analyze the significance of phenotype differences between allelic genotypes. The primer sequences of KASP markers are listed in Table S3.

### 2.6 Identification of candidate genes

To identify potential candidate genes associated with starch quality traits, the IWGSC online database (http://www.wheatgenome.org/) was used to search for all genes within the LD interval of significant and stable SNP markers (2 Mb upstream and 2 Mb downstream of the SNP flanking region). The UniProt protein database (https://www.uniprot.org/) and Ensembl Plants database (http://plants.ensembl.org/Triticum_aestivum/Gene) were utilized to predict the protein function of candidate genes. In the publicly available Expression Atlas database online (https://www.ebi.ac.uk/gxa/experiments/E‐GEOD‐38344/Results), RNA‐seq data from wheat endosperm and starch layers at 6, 9, and 14 days after flowering (Gillies et al., 2012) were used to study the expression characteristics of these genes. Based on functional annotation, genes that were highly expressed and genes that overlapped with stable SNPs were selected for further analysis. Quantitative real‐time polymerase chain reaction (qRT‐PCR) was used to analyze the expression of candidate genes in seeds of two extreme starch quality materials at 5, 10, 15, 20, 25, and 30 days after flowering. The two extreme starch quality materials are “Hongzhitou” and “Henong 326,” and their differences in starch quality are detailed in Table S4.

According to the manufacturer's instructions, total RNA was extracted from the leaves using TRIZOL reagent (Invitrogen), and reverse transcription was performed using the *PerfectStart* Uni RT&QPCR Kit (Transgen Biotech). The iCycler iQTM PCR detection system (Bio‐Rad) and *PerfectStart* Uni RT & qPCR Kit (TransGen Biotech) were used for qRT‐PCR with cDNA on a 96‐well plate with three replicates.

Wheat Actin (Genebank ID: LOC123114174) was used as a reference gene to calculate the relative quantification of gene expression using the delta Ct method. The gene IDs and primer sequences are detailed in Table S5.

### 2.7 Statistical analysis

The analysis of variance across multiple environments was conducted using R software.

The mean value of the traits was calculated using the best linear unbiased prediction (BLUP) method (Henderson, 1975) with the R software package lme4 (Bates et al., 2014).

Broad‐sense heritability (*h* ^2^) was estimated by variance components as follows: *h* ^2^ = *σ* ^2^G/(*σ* ^2^G + *σ* ^2^GE/E + *σ* ^2^e/*rE*), where *σ* ^2^G represented genetic variance, *σ* ^2^GE represented genotype × environmental interaction variance, *σ* ^2^e represented residual variance, *E* represented environmental quantity, and *r* represented the number of repetitions per material (Marcotuli et al., 2016).

Variance analysis and Pearson correlation analysis among phenotypic traits were performed using SPSS 22 (http://www.brothersoft.com/ibm‐spss‐statistics‐469577.html).

Manhattan and *Q*–*Q* plots were created using the qqman package in R software (Turner, 2018).

Gene expression heatmap was created using TBtools (C. Chen et al., 2023), and bar charts were drawn using Origin 8.0.

## 3 RESULTS

### 3.1 Phenotypic analysis of starch quality traits

Except for PV, which showed no significant differences between environments and years, and SB and FN, which showed no significant differences between genotypes, all other starch quality traits exhibited extremely significant differences among genotypes, environments, and years. Additionally, there were significant differences in the interaction between genotypes and environments for TV, as well as between genotypes and environments, and between genotypes and years for PT and T (Table S6). The variation ranges of PV, TV, BD, FV, SB, PT, T, FN, and DS in different environments were 2429–4061, 1672–2975, 265–1501, 2869–4251, 994–1797, 5.8–6.87, 62.75–88.05, 187.00–1123.00, and 4.70–8.36, respectively. The ranges of variation coefficients were 5.89%–7.23% for PV, 5.07%–6.85% for TV, 15.49%–23.66% for BD, 3.69%–5.70% for FV, 6.01%–8.21% for SB, 2.09%–2.53% for PT, 1.43%–9.68% for T, 16.62%–23.47% for FN, and 6.21%–7.38% for DS, respectively. The *h* ^2^ values of starch quality traits ranged from 0.45 to 0.87 (Table 1). Except for T, starch quality traits based on BLUP values were approximately normally distributed (Figure S1). Correlation analysis was conducted on starch quality traits based on BLUP values from four environments (Table 2). Significant correlations were found among starch quality traits, with the strongest correlation observed between PV and TV (*r* = 0.748), followed by the correlation between PV and BD (*r* = 0.679).

**TABLE 1:** Phenotype variations and heritability of starch quality traits.

Trait	Environment	Minimum	Maximum	Mean	SD	CV (%)	*h* ^2^
PV	2020EM	2330	3962	3155.34	226.25	7.17	0.77
	2020QT	2429	4061	3221.67	231.36	7.18	
	2021EM	2717	3717	3227.85	190.07	5.89	
	2021QT	2492	3940	3148.23	227.58	7.23	
TV	2020EM	1672	2724	2226.71	152.62	6.85	0.63
	2020QT	1795	2975	2362.41	154.69	6.55	
	2021EM	1991	2681	2350.24	119.20	5.07	
	2021QT	1984	2800	2407.71	141.24	5.87	
BD	2020EM	457	1479	931.38	166.38	17.86	0.82
	2020QT	265	1501	856.72	199.09	23.24	
	2021EM	472	1239	871.25	134.95	15.49	
	2021QT	282	1276	739.74	174.99	23.66	
FV	2020EM	2869	4251	3563.46	203.13	5.70	0.63
	2020QT	3007	4398	3747.66	181.42	4.84	
	2021EM	3388	4192	3775.67	139.23	3.69	
	2021QT	3109	4229	3658.98	170.32	4.65	
SB	2020EM	1054	1707	1333.01	104.26	7.82	0.37
	2020QT	1068	1797	1379.46	113.20	8.21	
	2021EM	1183	1643	1423.82	85.56	6.01	
	2021QT	994	1510	1250.04	99.50	7.96	
PT	2020EM	5.80	6.67	6.36	0.14	2.25	0.48
	2020QT	5.80	6.87	6.47	0.16	2.53	
	2021EM	6.07	6.73	6.40	0.13	2.09	
	2021QT	6.07	6.80	6.53	0.14	2.20	
T	2020EM	62.75	71.75	66.78	0.98	1.47	0.79
	2020QT	64.35	70.20	66.75	0.95	1.43	
	2021EM	64.35	88.05	68.90	6.67	9.68	
	2021QT	63.55	71.00	66.41	1.05	1.58	
FN	2020EM	187.00	644.00	395.41	81.41	20.59	0.45
	2020QT	246.00	738.00	485.75	87.69	18.05	
	2021EM	310.00	1123.00	722.97	169.65	23.47	
	2021QT	262.00	740.00	517.98	86.11	16.62	
DS	2020EM	5.18	7.86	6.59	0.46	6.97	0.87
	2020QT	5.49	8.36	6.91	0.49	7.06	
	2021EM	4.70	7.02	5.82	0.43	7.38	
	2021QT	5.47	7.84	6.71	0.42	6.21	

*Note*: 2020EM, 2020QT, 2021EM, and 2021QT represent the cropping seasons of 2019–2020 and 2020–2021 in Emin (EM) and Qitai (QT), respectively.

Abbreviations: BD, breakdown; CV, coefficient of variation; DS, damaged starch; FV, final viscosity; *h* ^2^, heritability; PT, peak time; PV, peak viscosity; SB, setback; SD, standard deviation; T, pasting temperature; TV, trough viscosity.

**TABLE 2:** Correlations (*r*) of the starch quality traits.

Trait	PV	TV	BD	FV	SB	PT	T	FN
TV	0.527^**^							
BD	0.679^**^	−0.206^**^						
FV	0.574^**^	0.748^**^	0.039					
SB	0.184^**^	−0.097^**^	0.303^**^	0.546^**^				
PT	0.038	0.648^**^	−0.515^**^	0.174^**^	−0.572^**^			
T	0.155^**^	0.037	0.138^**^	0.050	0.053	0.042		
FN	0.108^**^	0.293^**^	−0.132^**^	0.341^**^	0.168^**^	0.099^**^	0.016	
DS	−0.301^**^	−0.013	−0.338^**^	−0.095^**^	−0.153^**^	0.135^**^	−0.438^**^	−0.300^**^

Abbreviations: BD, breakdown; DS, damaged starch; FV, final viscosity; PT, peak time; PV, peak viscosity; SB, setback; T, pasting temperature; TV, trough viscosity.

**Significant difference at *p* < 0.01.

### 3.2 Genome‐wide association study

Based on BLUP values, GWAS identified 99 marker‐trait associations (MTAs) for starch quality traits (Figure S2; Table S7), including four for PV, seven for TV, 17 for BD, eight for FV, one for SB, seven for PT, 38 for T, 14 for FN, and three for DS. These MTAs were distributed on all chromosomes except 2A and 4D, explaining 4.58%–18.18% of the phenotypic variation (Table S7). Simultaneously, GWAS was performed in each environment, and a total of 34 significant and stable MTAs were detected, located on 31 loci (Table 3). This included one MTA for TV located on chromosome 5A; six for BD, all situated on chromosome 7A; three for FV, found on chromosomes 5A and 7A (2); two for PT, identified on chromosomes 5A and 6B; 12 for T, distributed across chromosomes 1A (3), 4A, 3B (2), 4B, 5B, 7B, 3D, 5D, and 7D; five for FN, located on chromosomes 3B, 4B, 7B (2), and 7D; and two for DS, situated on chromosomes 5D and 7D. The marker *5A_14411384* was significantly and stably associated with both TV and FV. The marker *5D_6525346* was significantly and stably associated with both T and DS. The markers *7A_327716996* and *7A_194287663* were significantly and stably associated with both BD and FV. These loci were pleiotropic (Table 3).

**TABLE 3:** Significant single‐nucleotide polymorphisms (SNPs) for starch quality traits were identified in multiple environments.

Trait	Multi‐environment	Marker	*p*‐value					*R* ^2^(%)				
			E1	E2	E3	E4	E5	E1	E2	E3	E4	E5
TV	E4 E5	*5A_14411384*				3.01E‐06	3.89E‐05				7.98	6.18
BD	E1 E2 E3 E4 E5	*7A_327716996*	1.89E‐06	1.98E‐07	9.20E‐07	5.15E‐05	2.89E‐09	8.27	9.63	8.97	6.02	12.30
	E1 E2 E3 E5	*7A_247105295*	4.46E‐07	1.62E‐08	4.14E‐06		1.07E‐09	9.21	11.27	7.96		12.96
		*7A_411628682*	6.47E‐05	3.65E‐06	5.38E‐05		8.67E‐07	5.98	7.74	6.26		8.59
		*7A_490494414*	1.73E‐06	1.02E‐07	7.92E‐06		5.47E‐09	8.32	10.06	7.54		11.88
	E2 E3 E5	*7A_194287663*		2.68E‐05	1.43E‐06		6.93E‐06		6.47	8.68		7.26
	E2 E5	*7A_518689480*		6.44E‐05			4.94E‐05		5.99			6.11
FV	E1 E2 E4 E5	*7A_327716996*	5.38E‐05	8.11E‐05		9.42E‐05	1.71E‐05	6.12	5.79		5.73	6.69
	E1 E4 E5	*7A_194287663*	1.54E‐05			4.58E‐05	8.04E‐06	6.92			6.19	7.17
	E4 E5	*5A_14411384*				3.94E‐07	1.82E‐08				9.32	11.11
PT	E1 E5	*5A_596659708*	2.95E‐05				1.88E‐05	6.50				6.63
	E4 E5	*6B_575943648*				1.78E‐06	5.26E‐06				8.20	7.44
T	E3 E4 E5	*5D_6525346*			3.85E‐12	1.39E‐08	4.86E‐13			17.39	1738.80	18.18
	E3 E4	*1A_155036542*			5.65E‐05	3.36E‐05				6.32	632.40	
	E3 E5	*1A_17254418*			9.19E‐05		6.73E‐07			5.83		8.73
		*1A_543073263*			2.78E‐06		1.54E‐08			8.11		11.17
		*3B_55132803*			1.08E‐05		2.45E‐05			7.22		6.45
		*3B_808668116*			8.89E‐05		3.43E‐06			6.18		8.14
		*3D_165412209*			6.50E‐06		5.71E‐06			7.60		7.45
		*4A_15432230*			1.79E‐05		3.99E‐06			6.90		7.59
		*4B_13221852*			5.56E‐05		4.75E‐05			6.16		6.04
		*5B_557502580*			3.74E‐05		3.22E‐08			6.42		10.69
T	E3 E5	*7B_35258345*			1.07E‐05		7.60E‐05			7.30		5.80
		*7B_36444528*			7.78E‐05		7.87E‐05			5.94		5.72
		*7D_7346444*			7.82E‐06		1.06E‐07			7.47		9.97
FN	E1 E4 E5	*7B_731193748*	2.98E‐05			5.15E‐05	4.31E‐06	6.44			6.04	7.53
	E3 E5	*7B_727265305*			5.65E‐05		6.88E‐06			6.18		7.26
	E4 E5	*3B_473235139*				4.30E‐05	2.66E‐05				5.09	5.34
		*4B_429701004*				3.94E‐05	9.30E‐06				5.13	5.95
		*7B_730229744*				5.61E‐05	6.49E‐06				5.96	7.27
		*7B_732577076*				1.43E‐05	3.71E‐05				6.94	6.30
		*7D_619458105*				7.35E‐05	2.29E‐05				4.76	5.42
DS	E1 E2 E3 E4 E5	*5D_6525346*	6.09E‐10	1.38E‐07	1.15E‐08	4.03E‐09	1.74E‐11	13.39	9.75	11.79	12.14	15.61
	E1 E5	*7D_625368658*	5.08E‐05				3.78E‐05	4.99				5.10

*Note*: E1, 2020EM; E2, 2020QT; E3, 2021EM; E4, 2021QT, which represent the cropping seasons in Emin (EM) and Qitai (QT) for the years 2019–2020 and 2020–2021, respectively; E5, BLUP, the best linear unbiased predictor of protein quality traits in 341 wheat accessions during two cropping seasons across two environments.

Abbreviations: BD, breakdown; DS, damaged starch; FV, final viscosity; PT, peak time; PV, peak viscosity; SB, setback; T, pasting temperature; TV, trough viscosity.

### 3.3 Haplotype analysis

**FIGURE 1:**
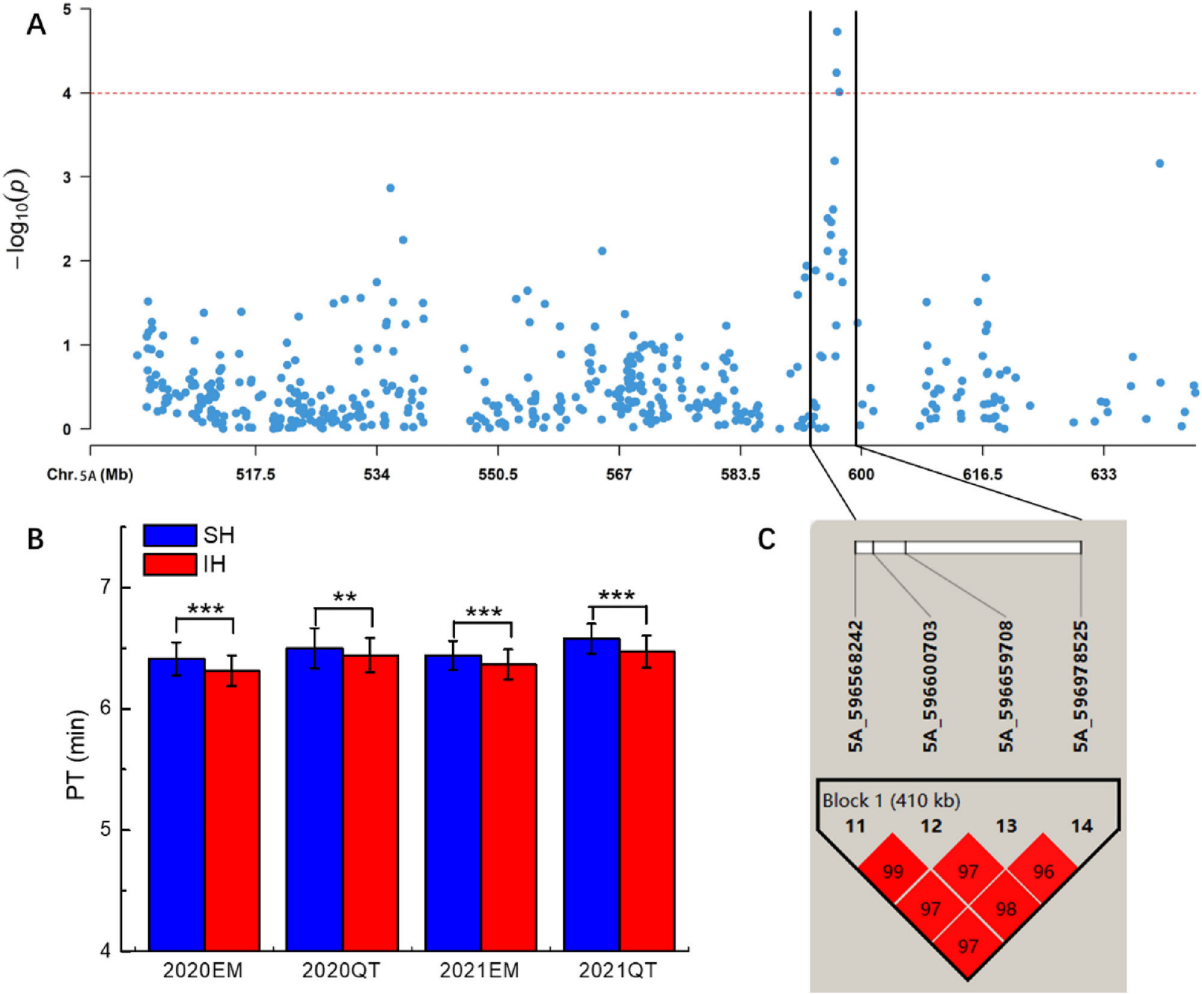
Haploid analysis of loci significantly correlated with peak time. (A) Manhattan plot for PT correlation interval; (B) phenotypic effects of haplotypes in block 1 in different environments; PT, peak time; SH, superior haplotype; IH, inferior haplotype; ***significant difference at *p* < 0.001; **significant difference at *p* < 0.01; 2020EM, 2020QT, 2021EM, and 2021QT represent the cropping seasons of 2019–2020 and 2020–2021 in Emin (EM) and Qitai (QT), respectively; (C) haplotype analysis of locus significantly associated with peak time in multiple environments.

The materials were grouped based on the genotypes of significant and stable association SNPs, and *t*‐tests were used to determine the significance of genotype effects on traits (Table S8). A SNP (*5A_596659708*) significantly associated with PT was identified on chromosome 5A. The PT differences among different alleles of this SNP reached extremely significant levels in four environments: 2020EM, 2020QT, 2021EM, and 2021QT (*p *< 0.01, Table S8). Haplotype analysis was conducted on the 2 Mb upstream and 2 Mb downstream of the SNP flank, and a 410 kb block was found at 596 Mb (Figure 1A,C). Four SNPs (*5A_596568242/5A_596600703/5A_596659708/5A_596978525*) were identified within this block. According to haplotypes, a total of 157 favorable haplotypes (AA/CC/AA/GG) and 112 unfavorable haplotypes (GG/TT/GG/TT) were classified. The *t*‐tests showed that the PT of favorable haplotypes was significantly higher (*p *< 0.01) than that of unfavorable haplotypes in the four environments of 2020EM, 2020QT, 2021EM, and 2021QT (Figure 1B).

### 3.4 KASP marker development and validation

**FIGURE 2:**
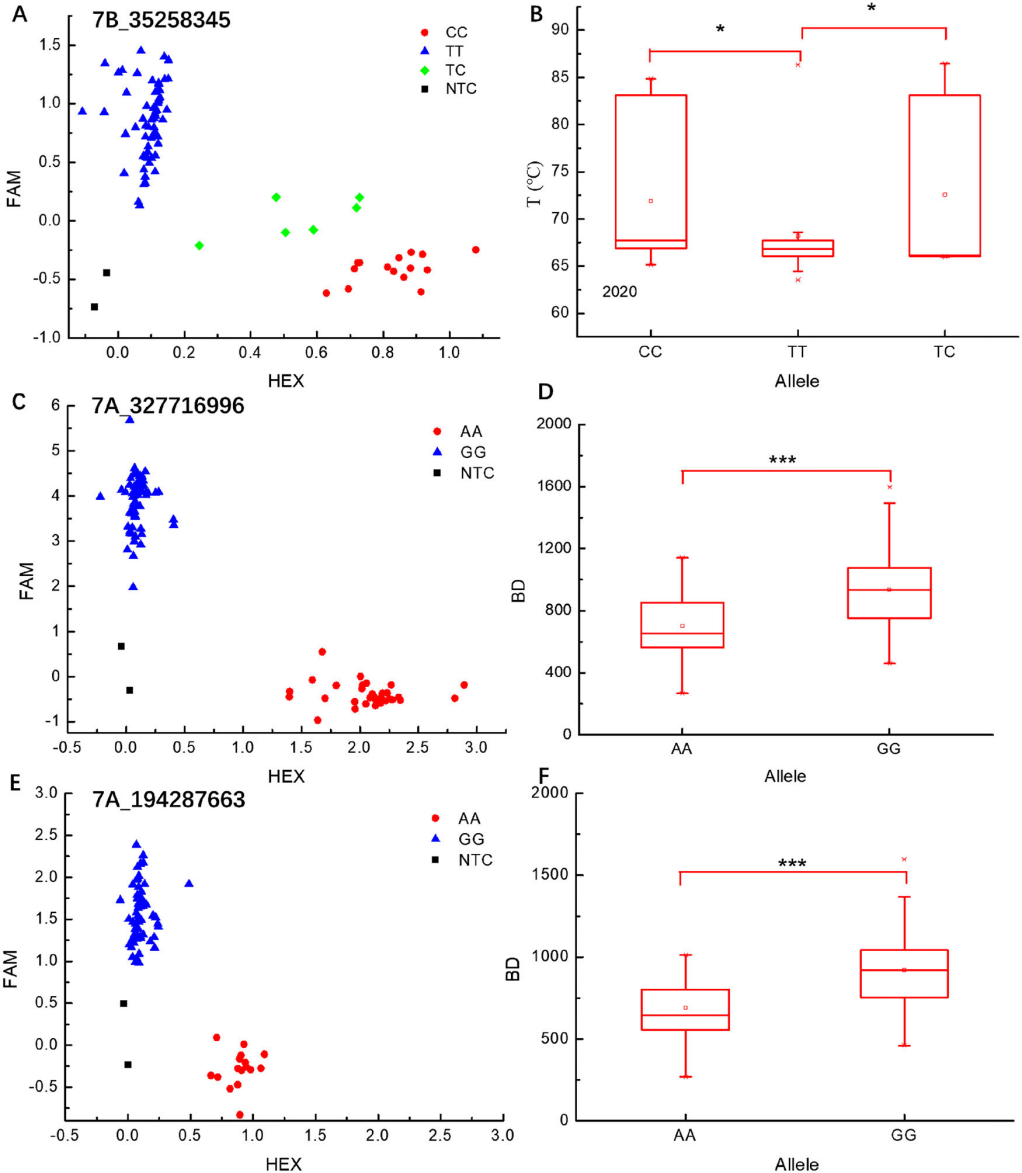
Kompetitive allele‐specific PCR (KASP) verification of a significant single‐nucleotide polymorphism (SNP) related to the starch quality. (A, C, and E) Scatter plots of KASP markers for T, BD, and BD, respectively; (B, D, and F) The variance of T, BD, and BD for accessions with different alleles; T, pasting temperature; BD, breakdown; red dots and blue triangles represent the homozygous alleles, green diamonds represent heterozygous alleles, and black squares on the bottom left of the plot indicate the no‐template control; ***significant difference at *p* < 0.001; *significant difference at *p *< 0.05.

Significant and stable association SNPs were selected to design KASP markers, and genotyping was conducted on randomly chosen subsets in the validation panel. The SNP marker *7B_35258345* showed a significant association with T (Table S8). The KASP marker developed for this SNP effectively differentiated the subsets based on alleles (Figure 2A). In 2020, the T of CC and TC alleles was notably higher than that of the TT allele (*p* < 0.05, Figure 2B). SNP markers *7A_327716996* and *7A_194287663* were significantly and stably associated with BD (Table S8). The KASP markers developed for these two SNPs effectively distinguished the subset based on alleles (Figures 2C,E). In 2020, BD was significantly higher in the GG allele compared to the AA allele (*p* < 0.001, Figures 2D,F).

### 3.5 Identification of candidate genes

Significantly and stably associated SNPs, which displayed notable phenotypic variations among different alleles across all four environments, were selected to search for candidate genes (Table S8). A total of 161 genes were identified within the 4 Mb sequences of these SNP flanks. Among them, the genes before and after SNPs, as well as the genes overlapping with SNPs, are detailed in Table S9. Thirty SNPs significantly and stably associated with starch quality were situated in the intergenic region, while four were located within genes. Gene ontology (GO) enrichment analysis revealed that these genes were involved in a total of 384 GO terms, categorized into 15 biological processes, one cellular component, and five molecular functions. The majority of genes are primarily involved in cellular processes and metabolic processes of biological processes, cellular anatomical entities of cellular components, binding, and catalytic activity of molecular functions (Figure S3C). Kyoto encyclopedia of genes and genomes analysis showed that these genes were mainly enriched in processes such as taurine and hypotaurine metabolism, endocytosis, fructose and mannose metabolism, peroxisome, cysteine and methionine metabolism, amino sugar and nucleotide sugar metabolism, and the plant mitogen‐activated protein kinase signaling pathway (Figure S3B).

**FIGURE 3:**
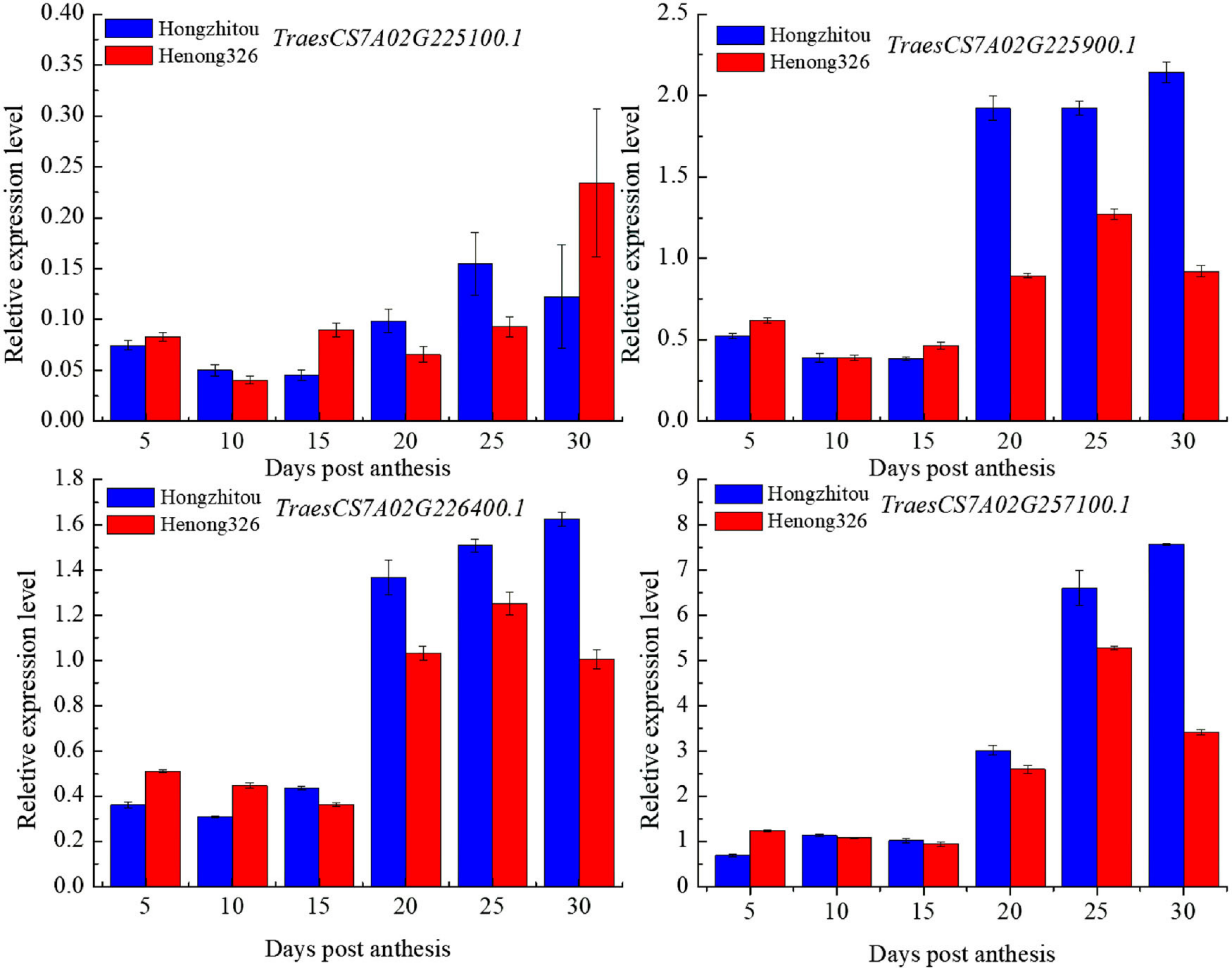
Expression of candidate genes in seeds of extreme starch quality materials at 5, 10, 15, 20, 25, and 30 days after flowering. HZT indicates Hongzhitou, and HN indicates Henong326.

Based on RNA‐seq data from public expression databases and their functional annotations and considering genes that overlap with significant SNPs, this study selected 13 candidate genes for qPCR analysis (Figure S3A). Four genes were differentially expressed in seeds of extreme starch quality materials (Figure 3; Table S10). *TraesCS7A02G225100.1* encodes the glycosyltransferase family 92 protein; *TraesCS7A02G225900.1* encodes a mitochondrial glycoprotein; *TraesCS7A02G226400.1* encodes the damage‐control phosphatase ARMT1‐like metal‐binding domain‐containing protein; and *TraesCS7A02G257100.1* encodes peptidylprolyl isomerase. These genes were all differentially expressed in the seeds of extreme starch quality materials at 20, 25, and 30 days after flowering. However, except for *TraesCS7A02G257100.1*, the expression levels of the other genes were not high.

## 4 DISCUSSION

### 4.1 Phenotypic variation and heritability of starch quality traits

Starch is the primary substance in wheat endosperm, providing the main source of carbohydrate energy for human daily life. Studying the genetic diversity of wheat starch quality is of great significance for the genetic improvement of starch quality (Matus & Hayes, 2002; Mourad et al., 2020). There were significant phenotype variations in starch quality traits of the association panel in this study (Table 1), consistent with previous research (Z. He et al., 2006; Muqaddasi et al., 2023; Ram & Sharma, 2013). Heritability provides the ability to pass on specific genetic traits to offspring (Ahmed et al., 2020). In this study, the heritabilities of PV, BD, T, and DS were all high (Table 1), consistent with the findings of Rahim et al. (2020). Continuous phenotype variation indicates that starch quality traits have polygenic inheritance effects. In this study, all phenotypic traits, except for T, were approximately normally distributed (Figure S1). In summary, this panel exhibited rich genetic variation and high levels of intraspecific inheritance, making it ideal for GWAS.

### 4.2 Genetic loci of RVA parameters

The MTA study established the relationship between genetic variation within the genome and specific phenotypes, ultimately detecting loci that support the corresponding traits. In previous research, loci associated with TV were identified on chromosomes 7A, 2B, 5B, and 7D (Tian et al., 2022); loci associated with BD were identified on chromosomes 1A, 3A, 4A, 6A, 1B, 3B, 4B, and 6D (Deng et al., 2014; Y. Zhang et al., 2009); loci associated with FV were identified on chromosomes 1A, 3A, 4A, 7A, 1B, 4B, 5B, 6B, 1D, 3D, 5D, and 6D (Batey et al., 2002; Deng et al., 2014; Sun et al., 2008; Y. Zhang et al., 2009); and a locus associated with PT was identified on chromosome 2A (Tian et al., 2022). This study identified one significant and stable locus associated with TV at 14 Mb on chromosome 5A; six significantly and stably loci associated with BD at 194 Mb, 247 Mb, 327 Mb, 411 Mb, 490 Mb, and 518 Mb on chromosome 7A; three significantly and stably loci associated with FV on chromosomes 5A and 7A, which overlapped with the associated loci of TV and BD; two significantly and stably loci associated with PT at 596 Mb on chromosome 5A and 575 Mb on chromosome 6B (Table 3). The loci detected in this study differ from those found in previous QTL research and may represent new loci for starch gelatinization characteristics.

The *Wx‐B1* gene (*TRAESCS4A02G418200*, Chr4A, 688,097,145–688,100,962) encodes GBSS to increase PV and BD (Batey et al., 2002; Briney, et al., 1998; Ram & Sharma, 2013). Batey et al. (2002) used two DH populations to identify QTLs of FV near *Wx‐B1* on chromosome 4A. Other studies have also discovered QTLs for starch gelatinization characteristics near the *Wx‐B1* gene on chromosome 4A (Batey et al., 2002; Tian et al., 2022). There is a phosphorylase‐encoding gene *TRAESCS7D02G365900* located at 473,617,760–473,624,545 bp on chromosome 7D, which may be involved in starch synthesis and degradation (Mishra et al., 2016; Tetlow et al., 2004; Tickle et al., 2009). Tian et al. (2022) identified two SNP markers *(IAAV4275* and *Kukri_cp69088_774*) near this gene. However, this study did not find any loci related to starch quality traits in the vicinity of these regions.

This study identified 12 loci significantly and stably associated with T on chromosomes 1A, 4A, 3B, 4B, 5B, 7B, 3D, 6D, and 7D (Table 3). Some of these loci were consistent with previous research, which found QTLs associated with T on chromosomes 3A, 4A, 2B, 3B, 4B, 5B, 6B, 7B, 2D, 5D, and 6D (Deng et al., 2014; Tian et al., 2022; Zhao et al., 2009). According to reports, starch gelatinization characteristics are controlled by starch synthase (SS I and SS II) and GBSS I (Deng et al., 2014). However, this study did not identify any SNPs significantly associated with starch gelatinization characteristics near these genes. Previous studies had identified QTLs associated with PV on chromosomes 1A, 2A, 3A, 4A, 7A, 1B, 3B, 4B, 5B, 6B, 7B, 3D, 5D, and 6D (Deng et al., 2014; Sun et al., 2008; Udall et al., 1999; Y. Zhang et al., 2009), and QTLs associated with SB on chromosome 5A (Tian et al., 2022). However, this study did not identify any SNPs significantly and stably associated with PV and SB (Table 3). Based on the BLUP value, only one PV association locus was identified on chromosome 3A, and one SB association locus was identified on chromosome 7A (Table S7). Perhaps because the SNP chip and associated panel used in this study were different from previous studies, along with the possibility that the gene loci linked to starch gelatinization characteristics in earlier studies may not be polymorphic in the materials of this study, or their impact might be too subtle to be identified.

### 4.3 Genetic loci of FN

FN is one of the most crucial parameters for wheat flour quality, determining the baking quality grade of wheat and impacting the economic benefits of growers (Muqaddasi et al., 2023). This study identified significant and stable association loci with FN at 473 Mb on chromosome 3B, 429 Mb on chromosome 4B, 727–732 Mb on chromosome 7B, and 619 Mb on chromosome 7D (Table 3). Börner et al. (2018) detected four major QTLs for FN on chromosomes 5A, 7B, 4D, and 5D. The QTL on the long arm of chromosome 7B corresponds to the main gene locus controlling wheat late‐maturing alpha‐amylase. Mohler et al. (2014) identified eight, five, and three QTLs for FN in the Dream/Lynx, Bussard/W332‐84, and BAUB469511/Format populations, respectively. The QTL on the long arm of chromosome 7B was common to all three populations and consistently detected in each environment. This QTL shares a genomic region similar to the main QTL for alpha‐amylase content. This is also consistent with the identification of genetic regions associated with FN loci on chromosome 7B in this study (Table 3). Muqaddasi et al. (2023) identified two QTLs on 1A (360.5 Mb) and 5B (618.1 Mb), while Tang et al. (2017) detected 11 additive effect QTLs for FN on chromosomes 4A, 2B, 6B, 3D, 4D, and 7D, explaining 5.48%–31.91% of the phenotypic variation. Among them, the FN QTLs on chromosomes 4A, 6B, and 4D were stable. Martinez et al. (2018) utilized a 90K SNP Illumina iSelect array to conduct GWAS on 469 white winter wheat varieties and superior breeding lines, identifying nine loci associated with FN on chromosomes 4A, 5A, 7A, 7B, and 5D. J. Zhang et al. (2014) identified 13 QTLs for FN on chromosomes 2A, 5A, 7A, 1B, 2B, 3B, 4B, 6B, and 7B using both general linear model and MLM. The accumulation of these QTLs explained 45% of the phenotypic variation. Although the loci identified in this study on 3B, 4B, and 7D (Table 3) were located on the same chromosomes as in previous studies, they were not within the same genetic interval and may represent new loci for FN.

### 4.4 Genetic loci of DS

DS refers to the small starch granules separated from the main starch granules during the milling process of wheat. These smaller particles are more easily used by yeast for gas production, more prone to hydration, and more readily hydrolyzed by enzymes during dough preparation (S. Y. Liu et al., 2014; Mulla et al., 2010). The level of DS affects the FN in wheat (Ma et al., 2016), and in this study, the FN of wheat was significantly negatively correlated with DS (Table 2). The degree of starch damage depends on the hardness of wheat (Delcour and Hoseney, 1994). Hard grains require more grinding energy than soft grains to convert endosperm into flour. During the grinding process, more starch particles are physically damaged. The main difference in grain texture between soft wheat and hard wheat is determined by allelic differences in the puroindoline genes *Pina* and *Pinb* located on the 5DS chromosome (Bhave & Morris, 2008a, 2008b; Morris, 2002). Previous studies have shown that the grain texture *Pinb* gene, located on the 5DS chromosome, was the main QTL determining milling, hydration, and biscuit baking traits (Campbell et al., 2001). This study identified the marker *5D_6525346*, which was significantly and stably associated with DS, in the region approximately 4 Mb from *Pina* and *Pinb* (Table 3). The present research did not identify any closer SNPs, which may be related to the density and coverage of SNPs on chromosomes of the chips. Another significant and stably associated marker *7D_625368658* has not been reported in previous studies and may be a new locus of DS.

### 4.5 Development of KASP marker

It is not easy to improve the quality of wheat starch through phenotype selection in the early stages of breeding. Even in the later stages of breeding, starch quality is easily influenced by the environment. Therefore, in the breeding process, enhancing the quality of wheat starch through MAS is an effective method. By utilizing high‐density SNP markers across the entire genome, GWAS analysis has become a commonly used method to uncover the genetic components, providing valuable insights into the genetic structure of complex traits (Tian et al., 2022). KASP is employed to detect InDels or SNPs and is suitable for genotyping multiple SNP markers in various samples. This study selected SNPs significantly associated with starch quality traits identified by GWAS and converted them into KASP markers to validate the accuracy of GWAS results. The developed KASP markers can also be utilized for MAS breeding. This study successfully converted three KASP markers, one related to T and two related to BD. These markers classify the validation panel clearly, and the phenotypic differences among different alleles reached a significant level in 2020 (Figure 2).

### 4.6 Candidate gene identification

Wheat starch synthesis is influenced by a series of enzymes, including ADP glucose pyrophosphate synthase, sucrose synthase, GBSS, SS, starch branching enzyme (BE), and starch debranching enzyme (DBE) (Kumar et al., 2018). Some genes related to starch synthesis have been cloned, such as GBSS I, soluble starch synthase, BE, DBE, wax gene (Waxy), starch regulator enzyme (AGPase), and so forth (Guo et al., 2023; Tuncel & Okita, 2013). Some transcription factors are involved in regulating starch synthesis. Liu et al. (2023) investigated the role of the basic helix‐loop‐helix (bHLH) transcription factor TabHLH95 in starch synthesis. The Tabhlh95 knockout mutant showed smaller particle size and less starch content, while overexpression of Tabhlh95 enhanced starch accumulation and significantly increased 1000‐grain weight. This study identified four candidate genes that were differentially expressed in seeds of extreme materials at 20, 25, and 30 days after flowering. *TraesCS7A02G225100.1* encodes the glycosyltransferase family 92 protein. In Arabidopsis (*Arabidopsis thaliana* (L.) Heynh.), glycosyltransferase family 92 has three proteins, GALS1, GALS2, and GALS3, all of which are active β‐1,4‐galactan synthases (Ebert et al., 2018). However, the expression level of this gene was relatively low in seeds of winter wheat at different days after flowering. *TraesCS7A02G225900.1* encodes a mitochondrial glycoprotein, *TraesCS7A02G226400.1* encodes a damage‐control phosphatase ARMT1‐like metal‐binding domain‐containing protein, and *TraesCS7A02G257100.1* encodes a peptidylprolyl isomerase. These genes have not been reported in plants yet, and their effects on starch quality require further research.

## 5 CONCLUSION

The coefficient of variation for starch quality traits ranged from 1.43% to 23.66%, and the *h* ^2^ ranged from 0.37% to 0.87%. Significant correlations were observed among starch quality traits. Thirty‐four SNP markers significantly and stably associated with starch quality traits were identified, distributed across 31 loci. These include one locus for TV, six loci for BD, three loci for FV, one locus for SB, two loci for PT, 12 loci for T, five loci for FN, and two loci for DS. A 410 kb block related to PT was identified at 596 Mb on chromosome 5A. Successfully developed one KASP marker for T and two KASP markers for BD. Four candidate genes that might affect grain starch quality were identified. The research findings offer valuable insights for enhancing wheat quality traits through genetic improvement.

## AUTHOR CONTRIBUTIONS

**Yousheng Tian**: Writing—original draft; writing—review and editing. **Pengpeng Liu**: Data curation; investigation. **Xin Zhang**: Data curation. **Yichen Liu**: Investigation; validation. **Dezhen Kong**: Data curation; investigation. **Yingbin Nie**: Data curation; investigation. **Hongjun Xu**: Data curation; investigation. **Xinnian Han**: Data curation; investigation. **Wei Sang**: Project administration; resources. **Weihua Li**: Project administration; supervision.

## CONFLICT OF INTEREST STATEMENT

The authors declare no conflicts of interest.

## Supporting information



Table S1. The names of 341 accessions in the association panel.
**Table S2**. The names of 200 accessions in the validation panel.
**Table S3**. The KASP marker primer sequences.
**Table S4**. Protein quality traits of two accessions.
**Table S5**. The gene IDs and primer sequences used in real‐time quantitative polymerase chain reaction (RT‐qPCR).
**Table S6**. Analysis of variance for starch quality traits in 321 winter wheat.
**Table S7**. Significant SNPs associated with starch quality traits by genome‐wide association study using BLUP values.
**Table S8**. *P*‐values of *t*‐tests for efficacy of different alleles on starch quality traits.
**Table S9**. Genes nearby stable SNPs.
**Table S10**. Annotation of candidate genes.
**Figure S1**. Frequency distribution of BLUP values for starch parameters in 341 wheat cultivars.
**Figure S2**. Manhattan and quantile‐quantile (Q‐Q) plots for starch quality traits identified through genome‐wide association using BLUP values.
**Figure S3**. Analysis of candidate genes for starch quality traits.

## Data Availability

The data presented in the study are deposited in Figshare https://doi.org/10.6084/m9.figshare.26360404.
