# Supplementary material for: Genome‐wide association study and KASP marker development for starch quality traits in wheat
Source: Plant Genome. 2024 Sep 29;17(4):e20514. doi: 10.1002/tpg2.20514 (PMC11628900; doi:10.1002/tpg2.20514)
Supplement: Supplementary file 1 [file TPG2-17-e20514-s001.docx]

Table S1 The names of 341 accessions in the association panel

| Code | Name | Code | Name | Code | Name |
| --- | --- | --- | --- | --- | --- |
| 1 | Xindong 18 | 115 | 2013(86)/2-3-1 | 229 | YY-103/2 |
| 2 | Xindong 2 | 116 | 2013(86)/7-1-1 | 230 | YY-120/2 |
| 3 | Xindong 14 | 117 | 2013(94)/3-2-1 | 231 | YY-121/2 |
| 4 | Xindong 15 | 118 | 2013(94)/3-2-2 | 232 | YY-122/3 |
| 5 | Xindong 16 | 119 | 2013(98)/4-4-2 | 233 | YY-123/2 |
| 6 | Xindong 17 | 120 | 2013(104)/1-1-2 | 234 | YY-125/1 |
| 7 | Xindong 18 | 121 | 2013(104)/1-2-1 | 235 | YY-126/1 |
| 8 | Xindong 19 | 122 | 2013(108)/1-1-1 | 236 | YY-138/1 |
| 9 | Xindong 20 | 123 | 2013(110)/2-6-2 | 237 | YY-139/3 |
| 10 | Xindong 21 | 124 | 2013(127)/2-1-1 | 238 | YY-142/1 |
| 11 | Xindong 22 | 125 | 2013(130)/2-1-3 | 239 | YY-234/2 |
| 12 | Xindong 23 | 126 | 2013(130)/2-3-2 | 240 | M-3/4 |
| 13 | Xindong 24 | 127 | 2013(130)/2-4-1 | 241 | Shidong 03112 |
| 14 | Xindong 26 | 128 | 2013(133)/3-2-2 | 242 | Shidong 0358 |
| 15 | Xindong 27 | 129 | 2013(143)/1-3-2 | 243 | Shidong 0821 |
| 16 | Xindong 28 | 130 | 2013(148)/2-3-1 | 244 | Shidong 04104 |
| 17 | Xindong 29 | 131 | 2013(148)/5-2-2 | 245 | Shidong 01162 |
| 18 | Xindong 30 | 132 | 2013(154)/1-1-2 | 246 | Shidong 0349 |
| 19 | Xindong 31 | 133 | 2013(154)/4-1-1 | 247 | Shidong 0451 |
| 20 | Xindong 32 | 134 | 2013(158)/1-2-1 | 248 | Shidong 03194 |
| 21 | Xindong 33 | 135 | 2013(178)/1-4-1 | 249 | Shidong 06510 |
| 22 | Xindong 36 | 136 | 2013(178)/8-3-1 | 250 | JSH15-08 |
| 23 | Xindong 37 | 137 | 2013(183)/3-1-1 | 251 | JSH606 |
| 24 | Xindong 38 | 138 | 2013(190)/6-2-1 | 252 | JSH15-06 |
| 25 | Xindong 40 | 139 | 2013(202)/3-1-1 | 253 | Xingmu1602 |
| 26 | Xindong 41 | 140 | 2013(205)/5-1-1 | 254 | Xingmu 1603 |
| 27 | Xindong 42 | 141 | 2013(206)/2-1-1 | 255 | Fengyuandong 3 |
| 28 | Xindong 44 | 142 | 2013(223)/3-2-2 | 256 | 0858 |
| 29 | Xindong 45 | 143 | 2013(230)/6-4-2 | 257 | SDWW-7 |
| 30 | Xindong 48 | 144 | 2013(230)/8-2-1 | 258 | 03--40 |
| 31 | Xindong 49 | 145 | 2013(230)/10-1-3 | 259 | 08/16 |
| 32 | Xindong 51 | 146 | 2013(230)/11-2-1 | 260 | 04--1 |
| 33 | Xindong 52 | 147 | 2013(247)/5-1-1 | 261 | Xinniang 14-6151 |
| 34 | Xindong 53 | 148 | 2013(247)/5-4-2 | 262 | Xinniang 15-2217 |
| 35 | Xindong 54 | 149 | 2013(251)/1-1-1 | 263 | Xinniang 606 |
| 36 | Xindong 55 | 150 | 2013(251)/5-3-1 | 264 | 2015J/176 |
| 37 | Xindong 57 | 151 | 2013(255)/2-4-1 | 265 | HWX1109 |
| 38 | Xindong 60 | 152 | 2013(255)/6-1-1 | 266 | 09BC-3 |
| 39 | Shidong 7 | 153 | 2013(255)/8-4-2 | 267 | Zhonglumai 666 |
| 40 | Shidong 8 | 154 | 2013(255)/10-1-2 | 268 | JFM-2 |
| 41 | Shidong 9 | 155 | 2013(255)/11-2-1 | 269 | Xm1607 |
| 42 | Yinong 16 | 156 | 2013(255)/11-2-3 | 270 | CA15026 |
| 43 | Yinong 17 | 157 | 2013(255)/11-2-5 | 271 | BH5335 |
| 44 | Kuidong 4 | 158 | 2013(255)/11-2-7 | 272 | 0868 |
| 45 | Kuihua 1 | 159 | 2013(255)/11-3-2 | 273 | 0818 |
| 46 | 95（20）/8-1-2 | 160 | 2013(255)/11-8-2 | 274 | Xinniang 152148 |
| 47 | 99（55）/3-1-1 | 161 | 2013(255)/11-9-2 | 275 | Xinniang 152183 |
| 48 | 99（79）/2-1-2 | 162 | 2013(255)/12-2-8 | 276 | JHD06 |
| 49 | 2000(125)/4-2-1 | 163 | 2013(255)/12-5-1 | 277 | JHD09 |
| 50 | 2000（125）/4-2-2 | 164 | 2013(256)/1-2-3 | 278 | 2012J176 |
| 51 | 99AR142-1 | 165 | 2013(256)/4-1-1 | 279 | LFM1512 |
| 52 | 99AR144-1 | 166 | 2014(6)/9-1-2 | 280 | LFM1430 |
| 53 | 2009(6)/8-1-3 | 167 | 2014(13)/2-2-1 | 281 | 99-5091 |
| 54 | 2009(67)/3-3-1 | 168 | 2014(13)/5-1-2 | 282 | CA12107 |
| 55 | 2009(126)/10-2-1 | 169 | 2014(13)/5-1-5 | 283 | GZ1815 |
| 56 | 2009(172)/2-2-1 | 170 | 2014(14)/1-1-1 | 284 | GZ1820 |
| 57 | 2010(27)/6-5-1 | 171 | 2014(14)/6-1-2 | 285 | CA13012 |
| 58 | 2010(48)/1-2-1 | 172 | 2014(17)/10-1-2 | 286 | CA16125 |
| 59 | 2010(60)/4-4-3 | 173 | 2014(20)/8-1-2 | 287 | CA17097-1 |
| 60 | 2010(101)/4-2-3 | 174 | 2014(20)/11-2-1 | 288 | 16GZ1612 |
| 61 | 2010(112)/6-2-2 | 175 | 2014(35)/6-1-2 | 289 | Tangshan 6898 |
| 62 | 2010(126)/3-1-2 | 176 | 2014(37)/5-3-1 | 290 | Jimai 24 |
| 63 | 2010(162)/8-2-2 | 177 | 2014(71)/3-3-1 | 291 | Jimai 26 |
| 64 | 2010(229)/1-2-2 | 178 | 2014(73)/4-1-1 | 292 | Jimai 31 |
| 65 | 2011(65)/1-1-1 | 179 | 2014(73)/4-1-2 | 293 | Han 5316 |
| 66 | 2011(70)/4-1-4 | 180 | 2014(75)/8-2-1 | 294 | Han 6172 |
| 67 | 2011(275)/2-1-1 | 181 | 2014(75)/8-2-2 | 295 | Heniang 12 |
| 68 | 2012(27)/7-2-4 | 182 | 2014(75)/15-1-2 | 296 | Shixin 618 |
| 69 | 2012(27)/7-6-1 | 183 | 2014(85)/2-2-1 | 297 | Liangxing 66 |
| 70 | 2012(41)/1-2-2 | 184 | 2014(86)/5-2-3 | 298 | Liangxing 99 |
| 71 | 2012(80)/10-1-1 | 185 | 2014(94)/2-2-2 | 299 | Jimai 17 |
| 72 | 2012(82)/7-4-3 | 186 | 2014(95)/1-2-1 | 300 | Jimai 19 |
| 73 | 2012(84)/3-3-4 | 187 | 2014(123)/1-1-1 | 301 | Jimai 20 |
| 74 | 2012(84)/3-5-2 | 188 | 2014(123)/1-2-1 | 302 | Jimai 21 |
| 75 | 2012(103)/3-2-2 | 189 | 2014(131)/1-2-1 | 303 | Jimai 22 |
| 76 | 2012(104)/3-1-2 | 190 | 2014(135)/2-1-3 | 304 | Jimai 23 |
| 77 | 2012(107)/8-1-4 | 191 | 2014(151)/5-2-3 | 305 | Jimai 44 |
| 78 | 2012(111)/5-2-6 | 192 | 2014(156)/2-1-3 | 306 | Jimai 229 |
| 79 | 2012(162)/4-1-3 | 193 | 2014(174)/2-1-1 | 307 | Yannong 19 |
| 80 | 2012(214)/2-1-1 | 194 | 2014(180)/2-2-3 | 308 | Taishan 241 |
| 81 | 2012(17)/3-1-4 | 195 | 2014(184)/2-2-2 | 309 | Yannong 173 |
| 82 | 2012(41)/1-2-2 | 196 | 2014(184)/2-3-2 | 310 | Shannong 22 |
| 83 | 2012(68)/8-1-1 | 197 | 2014(186)/2-2-1 | 311 | Yan 1212 |
| 84 | 2012(80)/10-1-3 | 198 | 2014(191)/3-2-1 | 312 | Yumai 34 |
| 85 | 2012(82)/7-5-1 | 199 | 2014(191)/3-2-3 | 313 | Gaocheng 8901 |
| 86 | 2012(202)/4-1-2 | 200 | 2014(194)/14-2-1 | 314 | Zheng 366 |
| 87 | 2013(27)/8-5-1 | 201 | 2014(197)/1-2-2 | 315 | Zhengmai 379 |
| 88 | 2013(206)/2-1-1 | 202 | 2014(199)/1-2-1 | 316 | Zhengmai 7698 |
| 89 | 2011(70)/4-1-4 | 203 | 2014(200)/1-1-3 | 317 | Zhengmai 9023 |
| 90 | 2012(67)/9-1-2 | 204 | 2014(205)/10-1-2 | 318 | Zhoumai 18 |
| 91 | 2013(37)/5-4-1 | 205 | 2014(205)/10-2-2 | 319 | Zhoumai 19 |
| 92 | 2013(62)/2-5-1 | 206 | 2014(214)/2-1-2 | 320 | Zhoumai 21 |
| 93 | 2013(69)/4-1-2 | 207 | 2014(229)/7-1-1 | 321 | Zhoumai 22 |
| 94 | 2013(85)/2-1-1 | 208 | 2014(230)/7-2-2 | 322 | Zhoumai 27 |
| 95 | 2013(115)/1-5-1 | 209 | 2014(233)/2-1-1 | 323 | Zhoumai 28 |
| 96 | 2013(123)/1-1-1 | 210 | 2014(235)/5-1-1 | 324 | Zhoumai 30 |
| 97 | 2013(148)/4-1-2 | 211 | 2013(28)/8-1-1 | 325 | Zhoumai 32 |
| 98 | 2013(154)/2-3-1 | 212 | 2013(39)/4-1-1 | 326 | Fengdecunmai 5 |
| 99 | 2013(154)/4-1-3 | 213 | 2013(39)/5-2-2 | 327 | Guan 35 |
| 100 | 2013(178)/1-3-1 | 214 | 2013(39)/5-5-3 | 328 | Aikang 58 |
| 101 | 2013(223)/5-3-1 | 215 | 2013(39)/11-4-1 | 329 | Xiaoyan 22 |
| 102 | 2013(227)/1-1-1 | 216 | 2013(39)/15-1-1 | 330 | Xiaoyan 54 |
| 103 | 2013(227)/1-1-2 | 217 | 2013(39)/15-3-2 | 331 | Kemai 1 |
| 104 | 2013(230)/11-3-1 | 218 | FZ-35/2 | 332 | Xinong 979 |
| 105 | 2013(243)/5-2-1 | 219 | FZ-46/5 | 333 | Shinuan 02-1 |
| 106 | 2013(247)/5-2-1 | 220 | FZ-48/2 | 334 | Zhongyou 206 |
| 107 | 2013(248)/5-1-1 | 221 | FZ-50/4 | 335 | Zhongyou 9507 |
| 108 | 2013(13)/4-1-1 | 222 | FZ-59/1 | 336 | Y119 |
| 109 | 2013(23)/4-1-1 | 223 | YY-2/1 | 337 | Jing 411 |
| 110 | 2013(27)/8-4-1 | 224 | YY-22/4 | 338 | 03-6118 |
| 111 | 2013(27)/8-5-1 | 225 | YY-57/3 | 339 | compton/yangmai 158 |
| 112 | 2013(58)/7-2-1 | 226 | YY-58/1 | 340 | Libellula |
| 113 | 2013(69)/4-1-1 | 227 | YY-59/1 | 341 | Strampellula |
| 114 | 2013(79)/3-1-1 | 228 | YY-84/2 |  |  |

All of the accessions originate from different provinces in China.

Table S2 The names of 200 accessions in the validation panel

| Code | Name | Code | Name |
| --- | --- | --- | --- |
| DZ001 | Hongzhitou 10 | DZ312 | Heisuidongmai |
| DZ028 | Yinong 18 | DZ313 | Fengchan 3 |
| DZ029 | Yinong 19 | DZ314 | Shijiazhuang 54 |
| DZ030 | Yinong 20 | DZ317 | Jinmai 22 |
| DZ031 | Yinong 21 | DZ320 | Jingzuo 210 |
| DZ032 | Kuidong 4 | DZ322 | F42-7 |
| DZ034 | Kuihua 2 | DZ326 | Duli |
| DZ039 | 80-453 | DZ327 | Xiusidun |
| DZ040 | 85(1) | DZ329 | Botre |
| DZ041 | 86(46)/0-2-5-3-1 | DZ333 | Quzishi21323 |
| DZ043 | 87YF5 | DZ337 | 93-410 |
| DZ044 | 89(100) | DZ348 | 92-5 |
| DZ045 | 89-(20)-1-2 | DZ356 | Lanketa |
| DZ046 | 89(34) | DZ358 | Athas66 |
| DZ047 | 89(35) | DZ370 | Lanlixiaomai |
| DZ048 | 89-117 | DZ384 | Xiaoyan 54 |
| DZ049 | 89-20/2 | DZ386 | Zhongyin 85 |
| DZ050 | 89-44 | DZ411 | Shi 02-6207 |
| DZ051 | 89-813 | DZ419 | Bainong 68 |
| DZ052 | 91(28) | DZ444 | Lankao 906-4 |
| DZ053 | 91-8 | DZ445 | 5R624（Lr4/C9355） |
| DZ054 | 96254-1 | DZ447 | 5R623 |
| DZ055 | 99-5019 | DZ448 | 5R619（Lr38） |
| DZ056 | 0118 | DZ453 | Zhou 8425B |
| DZ058 | 05/2111 | DZ462 | Shi 02-5289 |
| DZ060 | Xindong 39 | DZ465 | 035303 |
| DZ061 | 06/6155 | DZ467 | 0138 |
| DZ062 | D04-L4 | DZ468 | Huaimai 0208 |
| DZ064 | Jintianshan 8 | DZ469 | Nongda 3291 |
| DZ068 | 98(110)/5-3-1 | DZ473 | Keyi 4058 |
| DZ069 | 98(101)/13-3 | DZ478 | Shannong 2149 |
| DZ071 | 96(60)/3-1-3 | DZ480 | CA0391-1 |
| DZ076 | 98(111)/5-2-2 | DZ487 | Shan 253 |
| DZ086 | 98(21)/2-2-3 | DZ494 | Jingdong 8 |
| DZ087 | 99(55)/5-2 | DZ500 | Shijiazhuang 8 |
| DZ089 | 98(78)/9-3-1 | DZ501 | Shimai 15 |
| DZ093 | 98(83)/2-1-1 | DZ506 | Shi 4185 |
| DZ095 | 98(107)/1-3-1 | DZ507 | Heng 5229 |
| DZ098 | 98(107)/3-1-3 | DZ515 | Zheng 9023 |
| DZ101 | 98(75)/2-1-2 | DZ519 | Jimai 17 |
| DZ111 | 98(76)/2-2-3 | DZ520 | Jimai 19 |
| DZ117 | 97(98)/1-1-3 | DZ521 | Jimai 20 |
| DZ118 | 97(83)/1-1-1 | DZ522 | Jimai 21 |
| DZ123 | 97(97)/4-2-3 | DZ523 | Jimai 22 |
| DZ130 | 97(24)/5-1-1 | DZ529 | Lunxuan 987 |
| DZ143 | 98(111)/5-2-1 | DZ536 | Luyuan 502 |
| DZ145 | 98(114)/1-1-1 | DZ537 | Yanda 1885 |
| DZ146 | 98(124)2-2-2 | DZ538 | Fengshou |
| DZ152 | 74-25/luofulin 10 | DZ539 | Beinong 2 |
| DZ153 | 75-149/80-25 | DZ541 | Zhongmai 9 |
| DZ154 | 75-144/Hengshui 8116 | DZ547 | Linfen 6010 |
| DZ155 | 75-144/Fengshou | DZ560 | Hengshui 8116 |
| DZ158 | Banong 1403 | DZ565 | Shan 7859 |
| DZ159 | [(170/Jingzuo210) ×80-25] F5 | DZ566 | Jinnong 207 |
| DZ168 | (80A×Gaokang1) F1/(Dao×Jiu)/75-149/Jinan 13 | DZ568 | You8134 |
| DZ169 | (Dao×Jiu)//75-149×9-142/506/80A-2/Jingai 1 | DZ570 | Henong 326 |
| DZ172 | (Xindong 14×Lov10/somon/75-144×Hengshui 8116)F2 | DZ573 | Jinan13 |
| DZ175 | 75-149/(Y83-E4/Honggou×2407) | DZ575 | Jinanhe124 |
| DZ182 | 80-25 | DZ592 | Rusattea |
| DZ185 | 2001（38）/1-2-6 | DZ593 | Asosan |
| DZ186 | 2002（85）/4-1-2 | DZ597 | Atom MY4310 |
| DZ190 | 2001(43)/2-1-4 | DZ598 | Arha MY4270 |
| DZ193 | 2003（28）/4-3-1 | DZ599 | Atgton MY4168 |
| DZ195 | 2003（29）/3-2-4 | DZ600 | Knteh MY6280 |
| DZ196 | 2003（36）/6-3-2 | DZ601 | F181-3 MY5585 |
| DZ199 | 2003（76）/3-11-2 | DZ602 | MY4348 Attnla |
| DZ203 | 2002（41）/4-4-3 | DZ649 | Nongda 212 |
| DZ210 | 2000(115)/2-1-2 | DZ659 | Zhongmai 175 |
| DZ211 | 2000(139)/1-1-1 | DZ680 | Nongda 3488 |
| DZ214 | 2000(8)/1-3-2 | DZ681 | Yumai 34 |
| DZ215 | 2000(16)/2-2-2 | DZ682 | Wanmai 52 |
| DZ217 | 2001(43)/9-2-3 | DZ683 | Xinmai 18 |
| DZ218 | Kendong 03(10) | DZ684 | Yanzhan 4110 |
| DZ221 | Kendong 01(38) | DZ704 | Xiaobaidongmai |
| DZ224 | 2005(1)/7-4-6 | DZ708 | Kuchebaidongmai |
| DZ232 | 2005(62)/15-4-3 | DZ717 | Kayimuhong |
| DZ234 | 2005(65)/7-2-1 | DZ726 | Kadong 4 |
| DZ242 | 2012Hanqu3 | DZ728 | Youpilaina 1 |
| DZ244 | 2012Hanqu5 | DZ729 | Xiaoe 186 |
| DZ247 | 2012Hanqu8 | DZ731 | Aodesa6 |
| DZ250 | 2012Hanqu11 | DZ732 | Xinwukelan 83 |
| DZ255 | 2012Wanqu4 | DZ733 | Xinwukelan 84 |
| DZ256 | 2012Wanqu5 | DZ734 | Aodesa3 |
| DZ257 | 2012Wanqu6 | DZ735 | Wumang 1 |
| DZ259 | 2012Wanqu8 | DZ737 | Afuleer |
| DZ264 | 2012Zaosheng 2(95022-8-1-7) | DZ738 | Zaoyangmai |
| DZ265 | 2006(114)/5-10-2 | DZ740 | Aoweisite |
| DZ266 | 2007Lun2/6-3-1 | DZ742 | Shanqian 2 |
| DZ269 | 2007(6)/13-5-2 | DZ743 | Gaojiasuo |
| DZ270 | 2007(8)/26-2-2 | DZ745 | Huabei 187 |
| DZ271 | 2007(16)/25-5-2 | DZ746 | Huabei 497 |
| DZ272 | 2007(26)/6-1-3 | DZ747 | Beijing 6 |
| DZ274 | 2007(32)/7-4-3 | DZ748 | Beijing 7 |
| DZ275 | 2007(37)/20-1-3 | DZ752 | Jinan 4 |
| DZ277 | 2007(44)/13-2 | DZ754 | Baiyoubao |
| DZ290 | Jihe 124 | DZ758 | Gongnong19 |
| DZ291 | Rusaika | DZ759 | Luofulin 10 |
| DZ292 | Aigton MY4168 | DZ760 | Luofulin 18 |
| DZ299 | Mianyang 31 | DZ761 | Luofulin 13 |
| DZ306 | Kuiyinnan 4 | DZ762 | Cangdong 5 |

All of the accessions originate from different provinces in China.

Table S3 The KASP marker primer sequences

| Marker | Probe | Sequence(5'-3') |
| --- | --- | --- |
| *7B_35258345* | FAM | GAAGGTGACCAAGTTCATGCTAGCTCCATCCCAAGAATTATCAAT |
| *7B_35258345* | HEX | GAAGGTCGGAGTCAACGGATTAGCTCCATCCCAAGAATTATCAAC |
| *7B_35258345* | Common | CTTGGTTCATGGTGGCATCG |
| *7A_327716996* | FAM | GAAGGTGACCAAGTTCATGCTCTCCTTGGTGCACGCATCG |
| *7A_327716996* | HEX | GAAGGTCGGAGTCAACGGATTCTCCTTGGTGCACGCATCA |
| *7A_327716996* | Common | AACATGCATGCAACCTGGTCA |
| *7A_194287663* | FAM | GAAGGTGACCAAGTTCATGCTAGAATATTCGTCCGACAGTCCG |
| *7A_194287663* | HEX | GAAGGTCGGAGTCAACGGATTAGAATATTCGTCCGACAGTCCA |
| *7A_194287663* | Common | TGGATGAGGGTTGGATTGCC |

Table S4 Protein quality traits of two accessions

| Variety | PV(cp) | TV(cp) | BD(cp) | FV(cp) | SB(cp) | PT(min) | T(℃) | DS(%) |
| --- | --- | --- | --- | --- | --- | --- | --- | --- |
| Hongzhitou | 3635 | 2882 | 753 | 4654 | 1772 | 6.47 | 66.1 | 5.05 |
| Henong326 | 3786 | 2325 | 1461 | 4025 | 1700 | 6.33 | 84.85 | 2.35 |

PV, Peak viscosity; TV, Trough viscosity; BD, Breakdown; FV, Final viscosity; SB, Setback; PT, Peak time; T, Pasting temperature; DS, Damage starch

Table S5 The gene IDs and primer sequences used in real-time quantitative polymerase chain reaction (RT-qPCR)

| Gene ID | Primer F (5'-3') | Primer R (5'-3') |
| --- | --- | --- |
| *TraesCS7A02G223800.1* | GCTCAATCCAGCAGCTTTGTC | TGGAGTAAGCTCGCTCATGC |
| *TraesCS7A02G225100.1* | CCACCGCAGTATCCTTCTCC | CAAGGTTGACGCCTTTGTCG |
| *TraesCS7A02G225800.1* | AGAGCAAAGCTTACAGGTGGT | GCGATCGGTGTCCTCATCAA |
| *TraesCS7A02G225900.1* | GGGCCACAGTTCTCTGATCT | GTACTCGTGCAGCCAGTCAT |
| *TraesCS7A02G226000.1* | CAGGGTTGGTGTCGTCTTCA | TGATTTGGTCCCTGGTTCGG |
| *TraesCS7A02G226400.1* | GGACGAGGAGAACGCTAAGG | GCTGTGCAGACCCTAAGTCA |
| *TraesCS7A02G257100.1* | GACTGATGAAGAGGCGGTCA | ACGACAGCATCCTGCTTTGA |
| *TraesCS7A02G299800.1* | GTTCCCTAAGGACACGGCAA | TCTTCATGCACCCAGCCTTT |
| *TraesCS7A02G335400.1* | TGACTGGCGATTGTGCTGTT | GGCCAGGAGCTCTAGCAATC |
| *TraesCS7A02G336100.1* | ACAACGACATTGCTTCGCTG | GCAAGACCTTCAGACTGCG |
| *TraesCS7A02G336400.1* | ACTGTATCGTGTTGGTGTGGA | GGACCAACTTGTATACCTTCTGT |
| *TraesCS7B02G473800* | CATGTCAGCATCTGGACCGA | TGCTGATACCCGAAGCACTC |
| *Actin* | GGAAAAGTGCAGAGAGACACG | TACAGTGTCTGGATCGGTGGT |

Table S6 Analysis of variance for starch quality traits in 341 winter wheat

| Source of variance | *df* | Sum of squares | | | | | | | | |
| --- | --- | --- | --- | --- | --- | --- | --- | --- | --- | --- |
|  |  | PV | TV | BD | FV | SB | PT | T | FN | DS |
| Genotypes | 340 | 116650^***^ | 42418^***^ | 78000^***^ | 67532^***^ | 18325 | 0.04^***^ | 30.73^***^ | 27218 | 0.62^***^ |
| Environments | 1 | 7109 | 3266627^***^ | 3567382^***^ | 460308^***^ | 1310876^***^ | 4.518^***^ | 189.48^***^ | 1055803^***^ | 118.46^***^ |
| Years | 1 | 92 | 2424476^***^ | 2622125^***^ | 1250980^***^ | 128359^**^ | 0.754^***^ | 57.03^***^ | 10563095^***^ | 77.11^***^ |
| Genotypes×Environments | 339 | 27312 | 15189^**^ | 12894 | 18601 | 8334 | 0.017^**^ | 8.29^***^ | 8717 | 0.08 |
| Genotypes×Years | 339 | 24518 | 12720 | 13490 | 20760 | 9291 | 0.016^**^ | 6.78^***^ | 8194 | 0.06 |
| Residual | 332 | 27665 | 11205 | 11470 | 38249 | 17613 | 0.012 | 4.51 | 28431 | 0.12 |

PV, Peak viscosity; TV, Trough viscosity; BD, Breakdown; FV, Final viscosity; SB, Setback; PT, Peak time; T, Pasting temperature; DS, Damage starch；*** indicate significant differences at *P* < 0.001, ** indicate significant differences at *P* < 0.01.

Table S7 Significant SNPs associated with starch quality traits by genome-wide association study using BLUP values

| Trait | Marker | Chromosome | Position(bp) | *P*-value | *R^2^*(%) |
| --- | --- | --- | --- | --- | --- |
| PV | *3A_730209978* | 3A | 730209978 | 9.42E-05 | 5.61 |
|  | *3A_730618433* | 3A | 730618433 | 9.39E-05 | 5.62 |
|  | *3A_730663519* | 3A | 730663519 | 4.18E-05 | 6.12 |
|  | *3A_731641533* | 3A | 731641533 | 2.37E-06 | 6.79 |
| TV | *5A_14411384* | 5A | 14411384 | 3.89E-05 | 6.18 |
|  | *3B_60880348* | 3B | 60880348 | 5.77E-05 | 5.93 |
|  | *3B_61875433* | 3B | 61875433 | 3.72E-05 | 6.20 |
|  | *3B_61917217* | 3B | 61917217 | 4.47E-05 | 6.09 |
|  | *3B_62413754* | 3B | 62413754 | 4.73E-05 | 6.05 |
|  | *7B_605899749* | 7B | 605899749 | 2.72E-05 | 6.40 |
|  | *7B_611778464* | 7B | 611778464 | 4.58E-05 | 6.07 |
| BD | *3A_696210995* | 3A | 696210995 | 3.55E-05 | 6.27 |
|  | *7A_194287663* | 7A | 194287663 | 6.93E-06 | 7.26 |
|  | *7A_247105295* | 7A | 247105295 | 1.07E-09 | 12.96 |
|  | *7A_327716996* | 7A | 327716996 | 2.89E-09 | 12.30 |
|  | *7A_411628682* | 7A | 411628682 | 8.67E-07 | 8.59 |
|  | *7A_490494414* | 7A | 490494414 | 5.47E-09 | 11.88 |
|  | *7A_518689480* | 7A | 518689480 | 4.94E-05 | 6.11 |
|  | *5B_62914772* | 5B | 62914772 | 8.27E-05 | 5.70 |
|  | *5B_78318428* | 5B | 78318428 | 4.76E-05 | 6.06 |
|  | *5B_389475111* | 5B | 389475111 | 4.28E-05 | 6.29 |
|  | *5B_400478515* | 5B | 400478515 | 1.96E-05 | 6.81 |
|  | *5B_402708744* | 5B | 402708744 | 4.63E-05 | 6.07 |
|  | *5B_405705041* | 5B | 405705041 | 9.44E-05 | 5.62 |
|  | *5B_414653259* | 5B | 414653259 | 6.24E-05 | 5.88 |
|  | *5B_645235466* | 5B | 645235466 | 7.67E-05 | 5.75 |
|  | *6B_704875400* | 6B | 704875400 | 3.64E-05 | 6.34 |
|  | *1D_468571431* | 1D | 468571431 | 9.36E-05 | 5.63 |
| FV | *3A_727872751* | 3A | 727872751 | 2.36E-05 | 6.49 |
|  | *5A_14411384* | 5A | 14411384 | 1.82E-08 | 11.11 |
|  | *5A_413055852* | 5A | 413055852 | 5.95E-05 | 5.91 |
|  | *5A_413161075* | 5A | 413161075 | 2.65E-05 | 6.41 |
|  | *6A_12287208* | 6A | 12287208 | 7.83E-05 | 5.74 |
|  | *7A_194287663* | 7A | 194287663 | 8.04E-06 | 7.17 |
|  | *7A_327716996* | 7A | 327716996 | 1.71E-05 | 6.69 |
|  | *3D_586406297* | 3D | 586406297 | 4.01E-05 | 5.12 |
| SB | *7A_646758302* | 7A | 646758302 | 6.41E-05 | 5.86 |
| PT | *5A_596600703* | 5A | 596600703 | 5.76E-05 | 5.93 |
|  | *5A_596659708* | 5A | 596659708 | 1.88E-05 | 6.63 |

Continued Table S7

| Trait | Marker | Chromosome | Position(bp) | *P*-value | *R^2^*(%) |
| --- | --- | --- | --- | --- | --- |
| PT | *5A_596978525* | 5A | 596978525 | 9.76E-05 | 5.60 |
|  | *3B_753725906* | 3B | 753725906 | 9.30E-05 | 5.63 |
|  | *6B_515718721* | 6B | 515718721 | 9.70E-05 | 5.60 |
|  | *6B_575943648* | 6B | 575943648 | 5.26E-06 | 7.44 |
|  | *2D_76760495* | 2D | 76760495 | 5.79E-05 | 5.93 |
| T | *1A_17254418* | 1A | 17254418 | 6.73E-07 | 8.73 |
|  | *1A_514544585* | 1A | 514544585 | 9.86E-06 | 7.23 |
|  | *1A_543073263* | 1A | 543073263 | 1.54E-08 | 11.17 |
|  | *3A_488102680* | 3A | 488102680 | 2.95E-05 | 6.33 |
|  | *3A_488200017* | 3A | 488200017 | 2.95E-05 | 6.33 |
|  | *4A_15432230* | 4A | 15432230 | 3.99E-06 | 7.59 |
|  | *5A_18538314* | 5A | 18538314 | 5.15E-05 | 5.99 |
|  | *5A_23968729* | 5A | 23968729 | 1.88E-05 | 6.62 |
|  | *5A_24063054* | 5A | 24063054 | 2.63E-05 | 6.41 |
|  | *5A_443218971* | 5A | 443218971 | 1.32E-05 | 6.84 |
|  | *6A_409815859* | 6A | 409815859 | 5.84E-05 | 5.91 |
|  | *6A_568177905* | 6A | 568177905 | 3.19E-05 | 6.29 |
|  | *7A_130633008* | 7A | 130633008 | 1.80E-05 | 6.64 |
|  | *1B_490455198* | 1B | 490455198 | 5.27E-05 | 5.97 |
|  | *1B_608376803* | 1B | 608376803 | 9.65E-05 | 4.58 |
|  | *2B_290221542* | 2B | 290221542 | 2.40E-05 | 6.47 |
|  | *2B_382925897* | 2B | 382925897 | 2.11E-05 | 6.65 |
|  | *3B_36372575* | 3B | 36372575 | 2.03E-05 | 6.57 |
|  | *3B_41218501* | 3B | 41218501 | 1.66E-05 | 7.62 |
|  | *3B_41228492* | 3B | 41228492 | 2.18E-05 | 6.52 |
|  | *3B_42166738* | 3B | 42166738 | 2.81E-06 | 8.73 |
|  | *3B_55132803* | 3B | 55132803 | 2.45E-05 | 6.45 |
|  | *3B_760526996* | 3B | 760526996 | 9.10E-06 | 7.14 |
|  | *3B_800317453* | 3B | 800317453 | 6.10E-05 | 5.90 |
|  | *3B_808668116* | 3B | 808668116 | 3.43E-06 | 8.14 |
|  | *4B_13221852* | 4B | 13221852 | 4.75E-05 | 6.04 |
|  | *4B_91731783* | 4B | 91731783 | 8.91E-05 | 5.64 |
|  | *5B_544937602* | 5B | 544937602 | 4.11E-05 | 6.13 |
|  | *5B_557502580* | 5B | 557502580 | 3.22E-08 | 10.69 |
|  | *7B_35258345* | 7B | 35258345 | 7.60E-05 | 5.80 |
|  | *7B_36444528* | 7B | 36444528 | 7.87E-05 | 5.72 |
|  | *7B_427693802* | 7B | 427693802 | 9.17E-05 | 5.71 |
|  | *7B_715576903* | 7B | 715576903 | 3.49E-05 | 6.24 |
|  | *3D_165412209* | 3D | 165412209 | 5.71E-06 | 7.45 |

Continued Table S7

| Trait | Marker | Chromosome | Position(bp) | *P*-value | *R^2^*(%) |
| --- | --- | --- | --- | --- | --- |
| T | *5D_6525346* | 5D | 6525346 | 4.86E-13 | 18.18 |
|  | *5D_280304002* | 5D | 280304002 | 3.04E-05 | 6.32 |
|  | *5D_323799924* | 5D | 323799924 | 2.04E-05 | 6.57 |
|  | *7D_7346444* | 7D | 7346444 | 1.06E-07 | 9.97 |
| FN | *3B_473235139* | 3B | 473235139 | 2.66E-05 | 5.34 |
|  | *4B_429701004* | 4B | 429701004 | 9.30E-06 | 5.95 |
|  | *7B_723487498* | 7B | 723487498 | 1.35E-05 | 6.81 |
|  | *7B_723719795* | 7B | 723719795 | 3.90E-06 | 7.60 |
|  | *7B_723754503* | 7B | 723754503 | 9.17E-06 | 7.06 |
|  | *7B_723891393* | 7B | 723891393 | 4.28E-05 | 6.25 |
|  | *7B_723931864* | 7B | 723931864 | 2.63E-05 | 6.40 |
|  | *7B_726388445* | 7B | 726388445 | 7.79E-05 | 6.27 |
|  | *7B_727265305* | 7B | 727265305 | 6.88E-06 | 7.26 |
|  | *7B_730229744* | 7B | 730229744 | 6.49E-06 | 7.27 |
|  | *7B_731193748* | 7B | 731193748 | 4.31E-06 | 7.53 |
|  | *7B_732577076* | 7B | 732577076 | 3.71E-05 | 6.30 |
|  | *6D_128080139* | 6D | 128080139 | 4.72E-05 | 4.99 |
|  | *7D_619458105* | 7D | 619458105 | 2.29E-05 | 5.42 |
| DS | *7B_439607032* | 7B | 439607032 | 9.13E-05 | 5.62 |
|  | *5D_6525346* | 5D | 6525346 | 1.74E-11 | 15.61 |
|  | *7D_625368658* | 7D | 625368658 | 3.78E-05 | 5.10 |

PV, Peak viscosity; TV, Trough viscosity; BD, Breakdown; FV, Final viscosity; SB, Setback; PT, Peak time; T, Pasting temperature; DS, Damage starch

Table S8 *P*-values of *t*-tests for efficacy of different alleles on starch quality traits

| Trait | Marker | Reference | Allele | | | Number | | | *P*-value | | | |
| --- | --- | --- | --- | --- | --- | --- | --- | --- | --- | --- | --- | --- |
|  |  |  |  |  |  |  |  |  | 2020EM | 2020QT | 2021EM | 2021QT |
| TV | *5A_14411384* | G | AA | GA | GG | 40 | 11 | 282 | 0.040 | 0.198 | 0.000 | 0.008 |
| BD | *7A_327716996* | C | CC | CT | TT | 217 | 18 | 99 | 0.000 | 0.000 | 0.000 | 0.000 |
|  | *7A_247105295* | G | GG | GT | TT | 222 | 53 | 59 | 0.000 | 0.000 | 0.000 | 0.000 |
|  | *7A_411628682* | G | AA | GA | GG | 59 | 55 | 220 | 0.000 | 0.000 | 0.000 | 0.000 |
|  | *7A_490494414* | G | AA | GA | GG | 92 | 25 | 217 | 0.000 | 0.000 | 0.000 | 0.000 |
|  | *7A_194287663* | C | CC | CT | TT | 259 | 11 | 64 | 0.000 | 0.000 | 0.000 | 0.000 |
|  | *7A_518689480* | G | CC | GC | GG | 113 | 18 | 200 | 0.000 | 0.000 | 0.000 | 0.000 |
| FV | *7A_327716996* | C | CC | CT | TT | 215 | 18 | 100 | 0.000 | 0.000 | 0.041 | 0.000 |
|  | *7A_194287663* | C | CC | CT | TT | 257 | 11 | 65 | 0.000 | 0.000 | 0.023 | 0.000 |
|  | *5A_14411384* | G | AA | GA | GG | 40 | 11 | 282 | 0.002 | 0.024 | 0.010 | 0.001 |
| PT | *5A_596659708* | G | AA | GA | GG | 174 | 13 | 146 | 0.000 | 0.003 | 0.000 | 0.000 |
|  | *6B_575943648* |  |  |  |  |  |  |  |  |  |  |  |
| T | *5D_6525346* | C | CC | CT | TT | 301 | 6 | 8 | 0.000 | 0.000 | 0.000 | 0.000 |
|  | *1A_155036542* | C | CC | CT | TT | 285 | 1 | 18 | 0.640 | 0.008 | 0.042 | 0.001 |
|  | *1A_17254418* | A | AA | AT | TT | 286 | 3 | 26 | 0.108 | 0.108 | 0.000 | 0.736 |
|  | *1A_543073263* | T | CC | TC | TT | 112 | 13 | 190 | 0.013 | 0.019 | 0.193 | 0.066 |
|  | *3B_55132803* | C | CC | CT | TT | 125 | 18 | 172 | 0.051 | 0.145 | 0.418 | 0.010 |
|  | *3B_808668116* | C | AA | CA | CC | 7 | 104 | 194 | 0.150 | 0.256 | 0.374 | 0.730 |
|  | *3D_165412209* | G | AA | GA | GG | 103 | 13 | 196 | 0.038 | 0.145 | 0.163 | 0.407 |
|  | *4A_15432230* |  |  |  |  |  |  |  |  |  |  |  |
|  | *4B_13221852* | G | AA | GA | GG | 223 | 22 | 81 | 0.012 | 0.178 | 0.541 | 0.420 |
|  | *5B_557502580* | G | GG | GT | TT | 293 | 2 | 20 | 0.003 | 0.463 | 0.000 | 0.017 |

Continued Table S8

| Trait | Marker | Reference | Allele | | | Number | | | *P*-value | | | |
| --- | --- | --- | --- | --- | --- | --- | --- | --- | --- | --- | --- | --- |
|  |  |  |  |  |  |  |  |  | 2020EM | 2020QT | 2021EM | 2021QT |
| T | *7B_35258345* | T | CC | TC | TT | 29 | 5 | 268 | 0.124 | 0.029 | 0.000 | 0.001 |
|  | *7B_36444528* |  |  |  |  |  |  |  |  |  |  |  |
|  | *7D_7346444* |  |  |  |  |  |  |  |  |  |  |  |
| FN | *7B_731193748* | G | CC | GC | GG | 113 | 46 | 175 | 0.000 | 0.022 | 0.000 | 0.000 |
|  | *7B_727265305* |  |  |  |  |  |  |  |  |  |  |  |
|  | *3B_473235139* |  |  |  |  |  |  |  |  |  |  |  |
|  | *4B_429701004* |  |  |  |  |  |  |  |  |  |  |  |
|  | *7B_730229744* | G | AA | GA | GG | 125 | 16 | 195 | 0.000 | 0.034 | 0.000 | 0.000 |
|  | *7B_732577076* |  |  |  |  |  |  |  |  |  |  |  |
|  | *7D_619458105* | A | AA | AG |  | 223 | 113 |  | 0.000 | 0.032 | 0.000 | 0.000 |
| DS | *5D_6525346* | C | CC | CT | TT | 308 | 7 | 22 | 0.000 | 0.000 | 0.000 | 0.000 |
|  | *7D_625368658* |  |  |  |  |  |  |  |  |  |  |  |

PV, Peak viscosity; TV, Trough viscosity; BD, Breakdown; FV, Final viscosity; SB, Setback; PT, Peak time; T, Pasting temperature; DS, Damage starch; 2020EM, 2020QT, 2021EM, and 2021QT represent the cropping seasons of 2019~2020 and 2020~2021 in Emin (EM) and Qitai (QT), respectively.

Table S9 Genes nearby stable SNPs

| Trait | Marker | Annotation | Candidate gene | |
| --- | --- | --- | --- | --- |
|  |  |  | Former | Latter |
| TV | *5A_14411384* | intergenic | *TraesCS5A02G018300* | *TraesCS5A02G018400* |
| BD | *7A_327716996* | intergenic | *TraesCS7A02G285100* | *TraesCS7A02G285200* |
|  | *7A_247105295* | intergenic | *TraesCS7A02G257500* | *TraesCS7A02G257600* |
|  | *7A_411628682* | intergenic | *TraesCS7A02G299700* | *TraesCS7A02G299800* |
|  | *7A_490494414* | intergenic | *TraesCS7A02G335200* | *TraesCS7A02G335300* |
|  | *7A_194287663* | downstream | *TraesCS7A02G224900* | |
|  | *7A_518689480* | intergenic | *TraesCS7A02G354100* | *TraesCS7A02G354300* |
| FV | *7A_327716996* | intergenic | *TraesCS7A02G285100* | *TraesCS7A02G285200* |
|  | *7A_194287663* | downstream | *TraesCS7A02G224900* | |
|  | *5A_14411384* | intergenic | *TraesCS5A02G018300* | *TraesCS5A02G018400* |
| PT | *5A_596659708* | upstream | *TraesCS5A02G405300* | |
|  | *6B_575943648* | intergenic | *TraesCS6B02G326300* | *TraesCS6B02G326500* |
| T | *5D_6525346* | intergenic | *TraesCS5D02G012300* | *TraesCS5D02G012400* |
|  | *1A_155036542* | intergenic | *TraesCS1A02G127500* | *TraesCS1A02G127600* |
|  | *1A_17254418* | intergenic | *TraesCS1A02G034600* | *TraesCS1A02G034700* |
|  | *1A_543073263* | intergenic | *TraesCS1A02G362500* | *TraesCS1A02G362600* |
|  | *3B_55132803* | intergenic | *TraesCS3B02G086900* | *TraesCS3B02G087000* |
|  | *3B_808668116* | intergenic | *TraesCS3B02G579100* | *TraesCS3B02G579200* |
|  | *3D_165412209* | intergenic | *TraesCS3D02G181900* | *TraesCS3D02G182000* |
|  | *4A_15432230* | intergenic | *TraesCS4A02G021500* | *TraesCS4A02G021600* |
|  | *4B_13221852* | intergenic | *TraesCS4B02G017900* | *TraesCS4B02G018000* |
|  | *5B_557502580* | intergenic | *TraesCS5B02G379300* | *TraesCS5B02G379400* |
|  | *7B_35258345* | intergenic | *TraesCS7B02G036400* | *TraesCS7B02G036500* |
|  | *7B_36444528* | intergenic | *TraesCS7B02G037200* | *TraesCS7B02G037300* |
|  | *7D_7346444* | intergenic | *TraesCS7D02G016600* | *TraesCS7D02G016700* |
| FN | *7B_731193748* | intergenic | *TraesCS7B02G475200* | *TraesCS7B02G475300* |
|  | *7B_727265305* | intergenic | *TraesCS7B02G470900* | *TraesCS7B02G471000* |
|  | *3B_473235139* | intergenic | *TraesCS3B02G294200* | *TraesCS3B02G294300* |
|  | *4B_429701004* | intergenic | *TraesCS4B02G201300* | *TraesCS4B02G201500* |
|  | *7B_730229744* | exonic | *TraesCS7B02G473800* | |
|  | *7B_732577076* | intergenic | *TraesCS7B02G476400* | *TraesCS7B02G476600* |
|  | *7D_619458105* | intergenic | *TraesCS7D02G522900* | *TraesCS7D02G523000* |
| SD | *5D_6525346* | intergenic | *TraesCS5D02G012300* | *TraesCS5D02G012400* |
|  | *7D_625368658* | intergenic | *TraesCS7D02G530900* | *TraesCS7D02G531000* |

PV, Peak viscosity; TV, Trough viscosity; BD, Breakdown; FV, Final viscosity; SB, Setback; PT, Peak time; T, Pasting temperature; DS, Damage starch

Table S10 Annotation of candidate genes

| ID | Start | End | Annotation |
| --- | --- | --- | --- |
| *TraesCS7A02G225100.1* | 194321593 | 194325575 | Glycosyltransferase family 92 protein |
| *TraesCS7A02G225900.1* | 195397282 | 195399922 | Mitochondrial glycoprotein |
| *TraesCS7A02G226400.1* | 195736631 | 195742118 | Damage-control phosphatase ARMT1-like metal-binding domain-containing protein |
| *TraesCS7A02G257100.1* | 246264792 | 246270837 | peptidylprolyl isomerase |

Figure S1 Frequency distributions of BLUP values for starch parameters in 341 wheat cultivars

PV, Peak viscosity; TV, Trough viscosity; BD, Breakdown; FV, Final viscosity; SB, Setback; PT, Peak time; T, Pasting temperature; DS, Damage starch

Figure S2 Manhattan and quantile-quantile (Q-Q) plots for starch quality traits identified through genome-wide association analysis using BLUP values

Continued Figure S2

A, B, C, D, E, F, G, H, I, Manhattan and Q-Q plots for peak viscosity, trough viscosity, breakdown, final viscosity, setback, peak time, pasting temperature, falling number, damaged starch, respectively; A horizontal line represents the significance threshold at which markers were considered associated with a trait (*P* < 1E-4, =4).

Figure S3 Analysis of candidate genes for starch quality traits

A, Heat map of candidate genes for starch quality traits (Data processed using log10); "L" indicates low pasting temperature, representing the variety as "Hongzhitou"; "H" indicates a high pasting temperature, representing the variety as "Henong 326"; 5, 10, 15, 20, 25, and 30 refer to the days after anthesis; B, KEGG pathway analysis of candidate genes for starch quality traits; C, GO secondary classification of candidate genes for starch quality traits.
